# Novel magnetic Fe_3_O_4_/g-C_3_N_4_/MoO_3_ nanocomposites with highly enhanced photocatalytic activities: Visible-light-driven degradation of tetracycline from aqueous environment

**DOI:** 10.1371/journal.pone.0237389

**Published:** 2020-08-14

**Authors:** Tianpei He, Yaohui Wu, Chenyang Jiang, Zhifen Chen, Yonghong Wang, Gaoqiang Liu, Zhenggang Xu, Ge Ning, Xiaoyong Chen, Yunlin Zhao

**Affiliations:** 1 Hunan Provincial Key Laboratory for Forestry Biotechnology, Central South University of Forestry and Technology, Changsha, China; 2 Hunan Research Center of Engineering Technology for Utilization of Environmental and Resources Plant, Central South University of Forestry and Technology, Changsha, China; 3 Hunan Urban and Rural Ecological Planning and Restoration Engineering Research Center, Hunan City University, Hunan, China; 4 International Education Institute, Hunan University of Chinese Medicine, Changsha, China; University of California Santa Barbara, California, USA

## Abstract

In the present work, a series of magnetically separable Fe_3_O_4_/g-C_3_N_4_/MoO_3_ nanocomposite catalysts were prepared. The as-prepared catalysts were characterized by XRD, EDX, TEM, FT-IR, UV-Vis DRS, TGA, PL, BET and VSM. The photocatalytic activity of photocatalytic materials was evaluated by catalytic degradation of tetracycline solution under visible light irradiation. Furthermore, the influences of weight percent of MoO_3_ and scavengers of the reactive species on the degradation activity were investigated. The results showed that the Fe_3_O_4_/g-C_3_N_4_/MoO_3_ (30%) nanocomposites exhibited highest removal ability for TC, 94% TC was removed during the treatment. Photocatalytic activity of Fe_3_O_4_/g-C_3_N_4_/MoO_3_ (30%) was about 6.9, 5, and 19.9-fold higher than those of the MoO_3_, g-C_3_N_4_, and Fe_3_O_4_/g-C_3_N_4_ samples, respectively. The excellent photocatalytic performance was mainly attributed to the Z-scheme structure formed between MoO_3_ and g-C_3_N_4_, which enhanced the efficient separation of the electron-hole and sufficient utilization charge carriers for generating active radials. The highly improved activity was also partially beneficial from the increase in adsorption of the photocatalysts in visible range due to the combinaion of Fe_3_O_4_. Superoxide ions (·O_2_^−^) was the primary reactive species for the photocatalytic degradation of TC, as degradation rate were decreased to 6% in solution containing benzoquinone (BQ). Data indicate that the novel Fe_3_O_4_/g-C_3_N_4_/MoO_3_ was favorable for the degradation of high concentrations of tetracycline in water.

## Introduction

Tetracycline (TC) has been widely used to treat bacterial infections in humans and animals over the past few decades [1]. Besides for medical applications, TCs are also employed as a supplement in animal husbandry to promote animal growth [2]. However, due to the widespread use of TC, TC residues could be frequently detected in various environmental matrices [3,4]. The residual TC in the environment would seriously threaten the ecosystem and public health [5]. In general, TC cannot be effectively removed by conventional wastewater treatment processes, such as biological treatment [6]. Therefore, new techniques are required to remove TC in water. Recently, photocatalytic assays have received a lot of intensive research interest worldwide due to its high efficiency and reliability, and have emerged as highly effective techniques for TC degradation from water [7]. Some photocatalysts have the function of degrading pollutants while Excellent antibacterial activity [8,9]. Common traditional photocatalysts, such as ZnO, TiO_2_ [10], have been confirmed can degrade TC under light irradiation. However, in practical applications, these photocatalysts couldn’t fully utilize solar energy, which causes them to be hindered in practical applications [11]. Therefore, the high efficient sunlight-driven photocatalysts are received lots of attention for the photocatalytic treatment of TC.

Graphitic carbon nitride (g-C_3_N_4_) has a strong visible-light response due to it easily produced electrons and holes under visible-light irritation [12]. Due to its advantages of low toxicity, low preparation cost and high stability, it has been applied to the removal of organic pollutants in water, which has aroused extensive research interest [13,14]. Unfortunately, g-C_3_N_4_ has low redox potential, and its photogenerated electron-hole pairs are easy to recombine [15]. These result in the limitation of its application as a self-sufficient semiconductor for the removal of contaminations by photodegradation [16]. Therefore, various methods have been evolved to enhance the photocatalytic activity of pure g-C_3_N_4_, including metal deposition [17,18], nonmetal doping [19], coupling with other materials [20], and using nano-sized structures [21]. By coupling g-C_3_N_4_ with other semiconductors to form a heterojunction structure, the shortcomings of high recombination rate of photogenerated electron-hole pairs of a single photocatalyst could be solved [22]. It should be noted that Z-scheme heterostructure formed by combining g-C_3_N_4_ with other semiconductors can efficiently separate the photogenerated electrons and holes, thereby improving the photocatalytic activity of g-C_3_N_4_ under visible light [23]. Yu et al., proposed a direct g-C_3_N_4_-TiO_2_ Z-scheme photocatalyst, which increased the photocatalytic activity by 2.1 times compared to pure TiO_2_ [24]. Hong et al., reported that the photocatalytic efficiency of a Z-scheme V_2_O_5_/ g-C_3_N_4_ heterojunction for the degradation RhB was as high as 7.3 and 13.0 times that of pure g-C_3_N_4_ and V_2_O_5_, respectively [25]. MoO_3_ is a semiconducting material with wide gap, stable crystal structure, and photochromic sensitive nature [26]. It has been regarded as a promising candidate to form hybrid photocatalyst due to its special energetic and electrical properties [23]. Previous studies confirmed that combining with MoO_3_, the photocatalitic activities of many photocatalyst, including TiO_2_ [27], CdS [28], and polyimides [29], could be improved greatly. The composites possessed excellent photocatalytic activities by hindering charge recombination and improving charge transfer processes. Recently, researchers found that combining MoO_3_ with g-C_3_N_4_ could produce Z-scheme photocatalyst. The photocatalytic performance was enhanced due to the suitable band gaps between the two semiconductors. Under light illumination, the photogenerated charge carrier can be efficiently separated and thus generated more reactive species [23,30]. However, most photocatalysts with high activity exist as nano-powders [31], and due to the small particle size, they can be easily dispersed in water thus couldn’t be separated effectively [32]. This characteristic makes it practically limited and prone to secondary pollution [33]. To overcome the above problem, some magnetic materials, such as Fe_3_O_4_ and CoFe_2_O_4_, have been achieved considerable attention [34,35]. Magnetic materials can transfer their magnetic properties to photacatalyst after being loaded, thus the photocatalyst can be separated effectively and easily from the treated solution using external magnetic field [36].

Spurred on by aforesaid information, after integration and envision, a novel ternary Z-scheme photocatalyst composites was presented combining g-C_3_N_4_ with MoO_3_ and Fe_3_O_4_. The aim of this study was to develop an efficient photocatalyst by combining the interfacial connection of g-C_3_N_4_ and MoO_3_ as well as the easy separation of magnetic materials. Their physical and chemical properties were investigated via a series of characterization. The TC-degrading ability of the prepared composites was studied. The influences of MoO_3_ content on the photocatalytic performance of the composite were evaluated. The possible mechanisms for the photocatalytic activity enhancement and the TC degradation were presented.

## 1. Experimental

### 1.1. Material preparation

The g-C_3_N_4_ was prepared by direct heating of melamine to 520 ^o^C for 3 h in a muffle furnace, and the resultant samples was milled into powder for further use.

The Fe_3_O_4_/g-C_3_N_4_ was prepared by the following steps: g-C_3_N_4_ was dispersed in ethanol/water (1:2) solution and then treated with an ultrasonic cleaner at 300 W for 6h to form an uniform solution with 62.5 mg/L g-C_3_N_4_. 20 ml of 175 mg/L FeCl_3_ and 20 ml of 68 mg/L FeCl_2_ added into 500 mL of the suspension of g-C_3_N_4_. The mixture was stirred and dispersed at 80 ^o^C for 30 min prior to the quick injection of 10 mL of ammonia solution. The resultant mixture was stirred at 80 ^o^C for another 30 min. The as-obtained precipitate was washed several times with ultrapure water and absolute alcohol before being dried in air at 80 ^o^C for further use. The resultant sample was named Fe_3_O_4_/g-C_3_N_4_.

AHM (Ammonium heptamolybdate tetrahydrate) was added into ultrapure water with a little acetic acid. The resultant solution was adjusted to pH 3.5 with 36% acetic acid and stored at 80 ^o^C for 12 h to obtain amount of white precipitation. The precipitation was washed by absolute ethanol for 5 times and consequently dried in air at 60 ^o^C for 12 h (designed as secondary ammonium molybdate). After the obtained sample was ground for 30 minutes, it was sintered at 500 ^o^C for 2 hours under the protection of nitrogen. The resultant sample was named MoO_3_.

The Fe_3_O_4_/g-C_3_N_4_/MoO_3_ nanocomposites were synthesized by calcination method. Secondary ammonium molybdate and Fe_3_O_4_/g-C_3_N_4_ were taken separately in mortars, grounded for 30 mins. Then the two samples were mixed and thoroughly grounded for another 30 mins before being sintered at 500 ^o^C for 2 h under nitrogen atmosphere. After being cooled, the product was obtained. Following the same synthesis route different weight percentage of Fe_3_O_4_/g-C_3_N_4_/MoO_3_ nanocomposites were obtained varying the wt% of secondary ammonium molybdate maintaining wt ratio 10, 20, 30 and 40 wt%. All the Fe_3_O_4_/g-C_3_N_4_/MoO_3_ composites were denoted as Fe_3_O_4_/g-C_3_N_4_/MoO_3_(10%), Fe_3_O_4_/g-C_3_N_4_/MoO_3_(20%), Fe_3_O_4_/g-C_3_N_4_/MoO_3_(30%), and Fe_3_O_4_/g-C_3_N_4_/MoO_3_(40%).

### 1.2. Characterization

The XRD patterns were obtained by a Bruker D8 Advance X-ray diffractometer with CuKα radiation, employing scanning rate of 0.02^o^/sec in the 2θ range from 5^o^ to 90^o^. Surface morphology was studied by JSM-7500F SEM, using an accelerating voltage of 5 kV. The purity and elemental analysis of the products were obtained by EDX on JSM-7500F SEM. The microstructures were investigated by a JEM-2100F TEM with an acceleration voltage of 200 kV. HRTEM was conducted on a JEM-2100F. The UV-Vis DRS was performed by an UV270 spectrophotometer, utilizing BaSO_4_ was the reflectance. The FT-IR spectra were studied by a Nicolet-iS10 instruction. XPS data was obtained by an Escalab 250Xi apparatus. The surface area and pore properties were estimated by BET and BJH models using the adsorption data collected by Micro for TriStar II Plus 2.02 apparatus at -196 ^o^C. Thermo-gravimetric analysis (TGA) was carried out on a STA 449F3 thermal analyzer with a heating rate of 10 ^o^C/min from room temperature to 1000 ^o^C in an air flow. The photoluminescence (PL) spectra were obtained by a Fls980 fluorescence spectrophotometer with an excitation wavelength of 380 nm. Magnetic properties were investigated using a MPMS.

### 1.3. Photocatalytic activity measurement

The capacity of the synthesized catalysts to photodegrade TC was performed by a photochemistry reaction instrument (YM-GHX-V, Shanghai Yuming Instrument Co. Ltd, China) with a 1000 W Xe lamp applied as visible light source, as shown in S1 Fig. In the reaction system, the reaction solution is packed in a quartz tube with a capacity of 50 ml, and the quartz tube is fixed at a distance of 2 cm from the light source. An optical power meter (OPT-1A, China) was used to measure the intensity of the experimental lamp to be 37.5 mW/cm^2^ (λ >400 nm). A water circulation system was utilized to keep the reaction system at 15 ^o^C. In each experiment, 10 mg of the photocatalyst was added into 50 mL of TC solution (40 mg/L). Prior to illumination, the reaction solution was treated in dark for 30 min to achieve adsorption-desorption equilibium. Every 30 minutes, 0.5 mL was sampled from the reaction solution and centrifuged immediately at 5000 rpm for 7 min. The TC concentration was determined based on absorbance at 355 nm by Nano Drop 2000 spectrophotometer.

### 1.4. Active species trapping measurement

Radical scavenge experiments was performed to verify the role of active substances in the degradation of TC. Ethylenediaminetetraacetate (EDTA-2Na, 1 mM), potassium dichromate (K_2_Cr_2_O_7_, 50 μM), isopropanol (IPA, 10 mM), and benzoquinone (BQ, 1 mM), were respectively applied as the trapping agent of h^+^, e^−^, ·OH, and ·O_2_^−^ [37,38].

## 2. Results and discussion

### 2.1. Photocatalyst characterization

**Fig 1:**
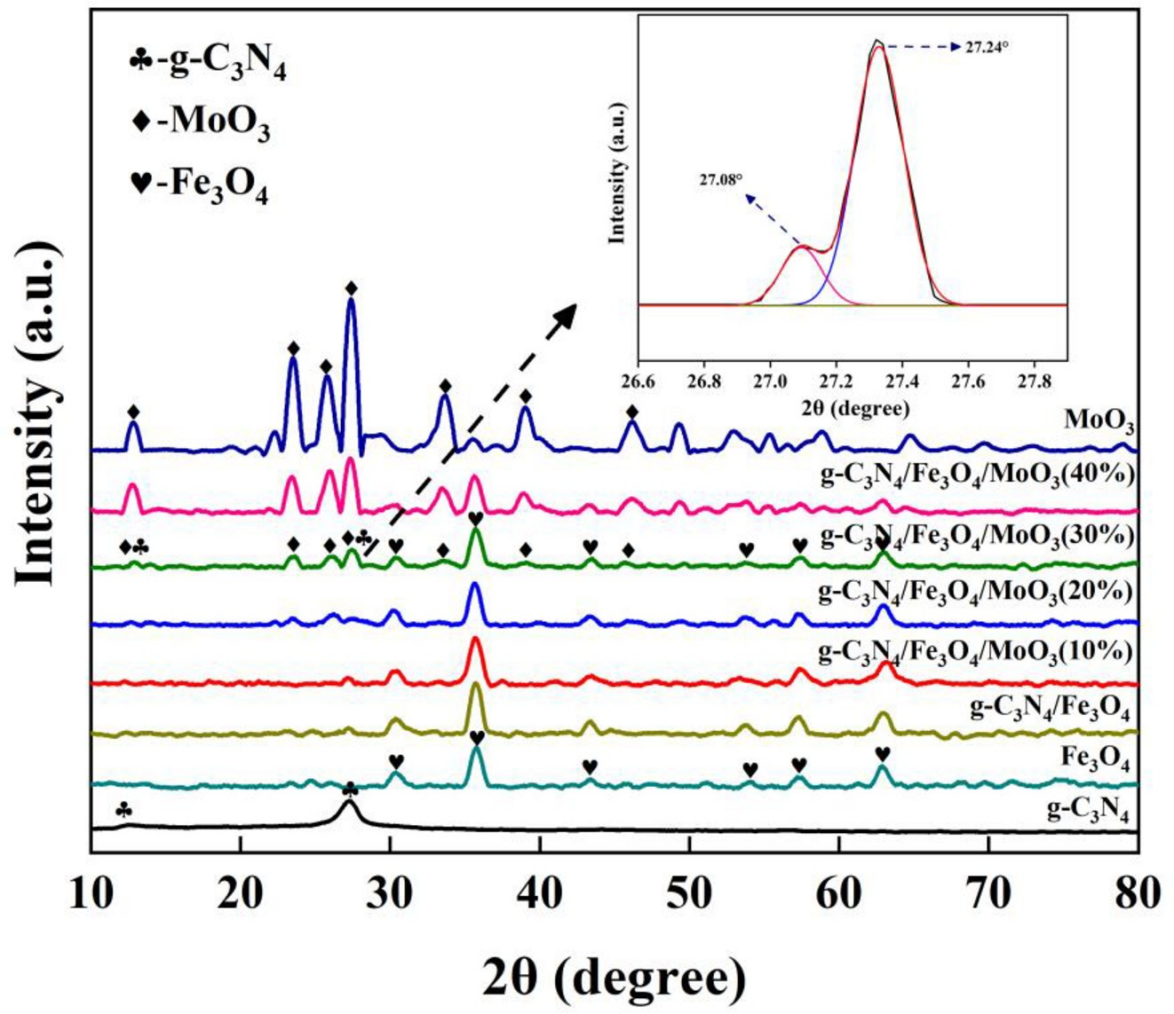
XRD patterns for the MoO_3_, g-C_3_N_4_, Fe_3_O_4_, Fe_3_O_4_/g-C_3_N_4_ and Fe_3_O_4_/g-C_3_N_4_/MoO_3_ nanocomposites. Inset image shows the deconvulation peaks for MoO_3_ and g-C_3_N_4_.

Fig 1 showed the typical XRD patterns of MoO_3_, g-C_3_N_4_, Fe_3_O_4_, Fe_3_O_4_/g-C_3_N_4_ and Fe_3_O_4_/g-C_3_N_4_/MoO_3_ composites. It could clearly observed that the (020), (110), (040), (021), (111), (060), and (200) peaks of MoO_3_ were at 12.83^o^, 23.46^o^, 25.76^o^, 27.40^o^, 33.75^o^, 39.07^o^, and 46.04^o^, which could be exactly indexed as the orthorhombic structure (α-MoO_3_) (JCPDF 35–0609) [39]. Previous study reported that MoO_3_ had three different crystalline structure, orthorhombic (α-MoO_3_), monoclinic (β-MoO_3_) and hexagonal (h-MoO_3_) and α-MoO_3_ was thermodynamically stable [40]. So it concluded that the proposed synthesis process benefit the growth of α-MoO_3_ which was more thermodynamically stable than β-MoO_3_. The (100) and (002) peaks of g-C_3_N_4_ appeared at 13.12^o^ and 27.52^o^, which were in consistent with the characteristic interplanar staking peaks of the inter-layer structural packing and aromatic systems, respectively [41]. The main peaks of Fe_3_O_4_ appeared at 35.83^o^, 43.18^o^, 53.17^o^, 57.43^o^ and 63.04^o^, well presented to the lattice plane (311), (400), (422), (511) and (440), respectively [42]. The Fe_3_O_4_/g-C_3_N_4_ nanocomposites had the peaks corresponding Fe_3_O_4_ and g-C_3_N_4_, indicting Fe_3_O_4_ were successfully deposited on g-C_3_N_4_ surface. The patterns for Fe_3_O_4_/g-C_3_N_4_/MoO_3_ nanocomposites were composed of the diffraction peaks corresponding to g-C_3_N_4_, MoO_3_ and Fe_3_O_4_, confirming the coexistence of the three materials. Moreover, it was clearly that the intensity of the peaks for MoO_3_ in Fe_3_O_4_/g-C_3_N_4_/MoO_3_ nanocomposites increased with the increase of the weight percent of MoO_3_. However, the peaks for g-C_3_N_4_ in the nanocomposites were not obviously observed as it overlapped with the peaks for MoO_3_. The inset XRD patters for Fe_3_O_4_/g-C_3_N_4_/MoO_3_ (30%) displayed the two deconvulation peaks at 27.32, suggesting the presence of both MoO_3_ and g-C_3_N_4_. These results further verified that Fe_3_O_4_ and g-C_3_N_4_ combined with MoO_3_ successfully.

**Fig 2:**
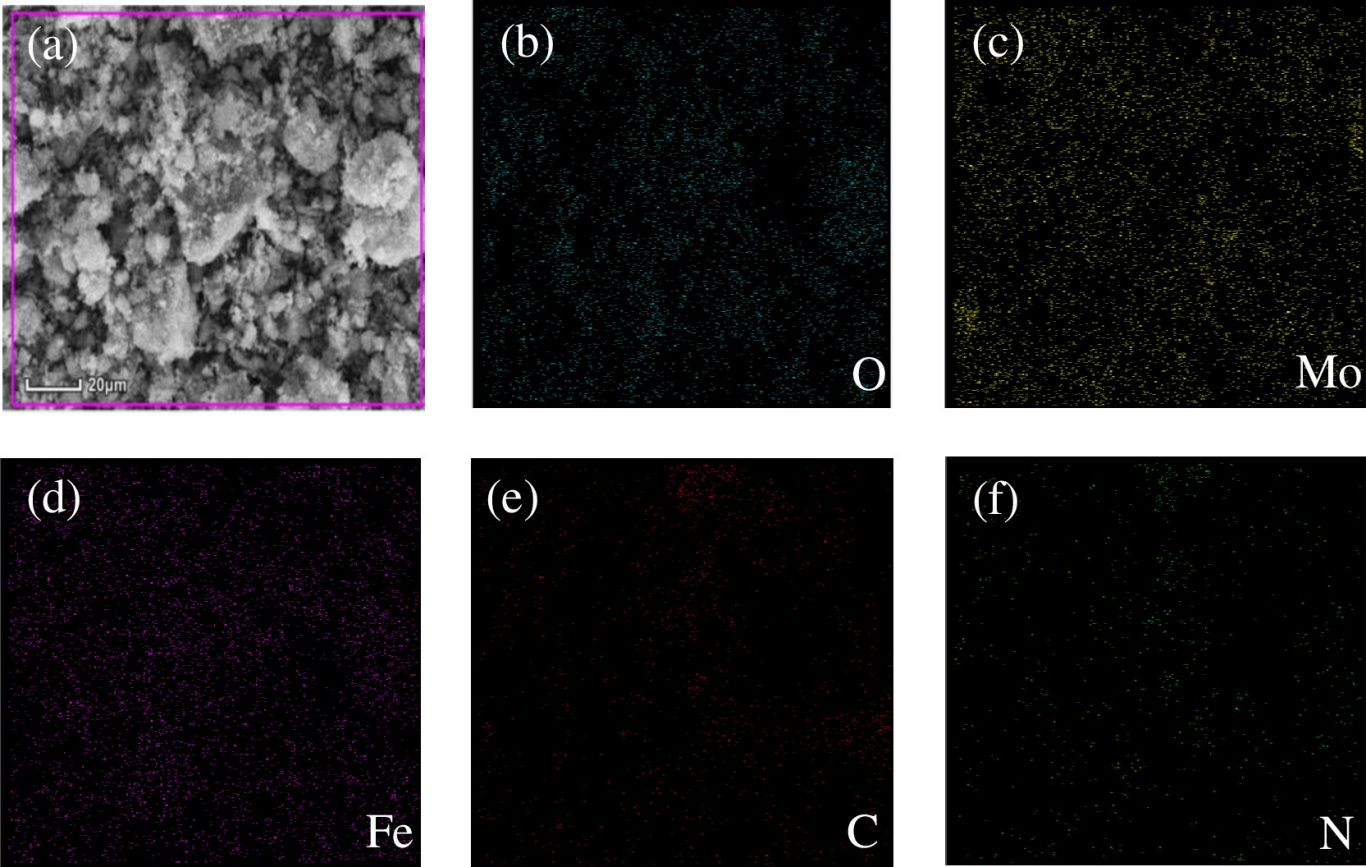
(a) SEM images of Fe_3_O_4_/g-C_3_N_4_/MoO_3_ (30%); (b-f) EDX mapping for the Fe_3_O_4_/g-C_3_N_4_/MoO_3_ (30%) nanocomposite.

Fig 2 exhibited the elemental mapping of the Fe_3_O_4_/g-C_3_N_4_/MoO_3_ (30%) nanocomposites which was detected from a randomly selected area of the nanocomposite using EDX detector. It could be clearly found C, N, Fe, O and Mo (Fig 2B–2F) were all homogeneous indicating uniform distributions of Fe_3_O_4_, g-C_3_N_4_, and MoO_3_ in the selected area of the corresponding SEM image (Fig 2A).

**Fig 3:**
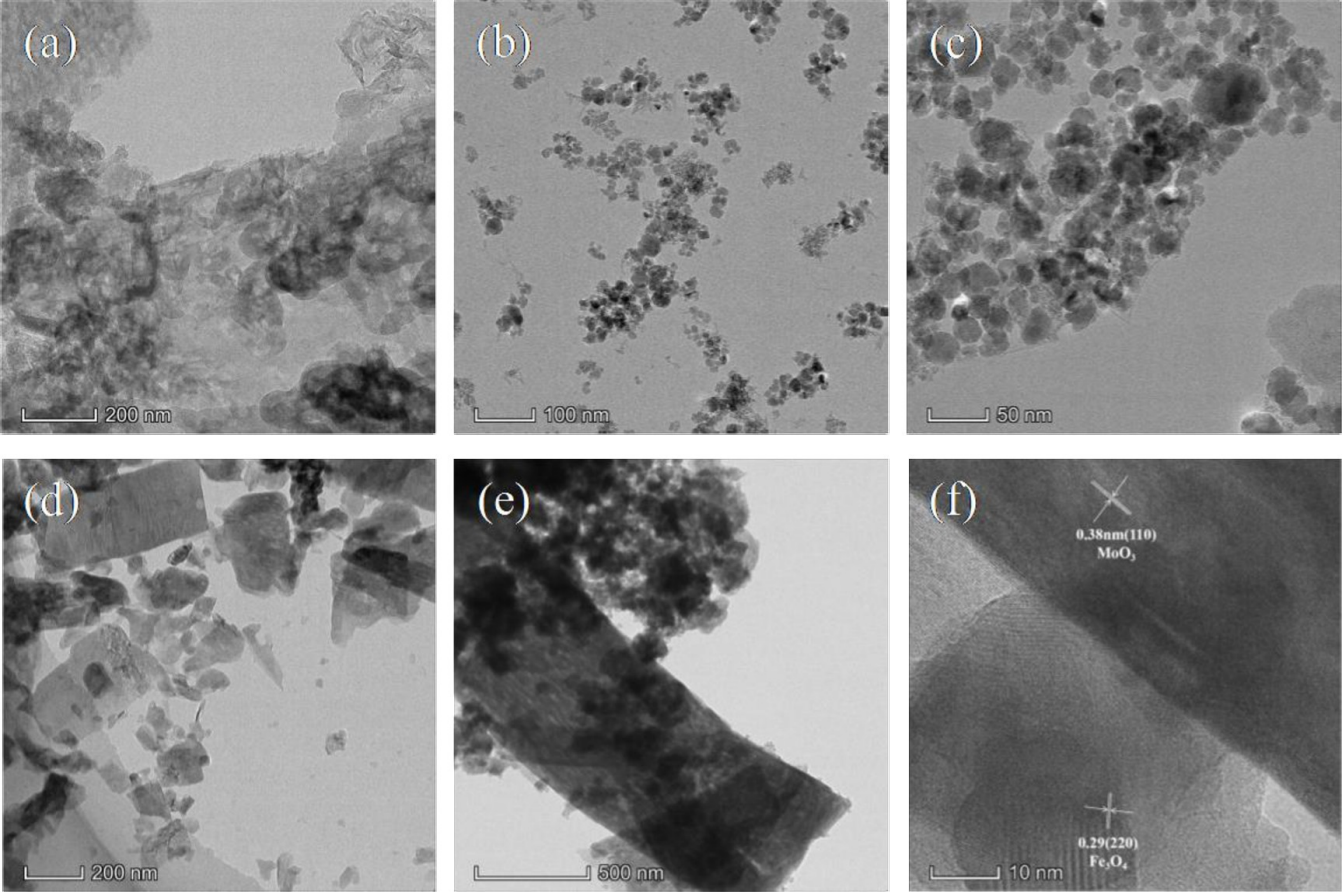
TEM of (a) g-C_3_N_4_; (b) Fe_3_O_4;_ (c) Fe_3_O_4_/g-C_3_N_4_; (d) MoO_3_; (e) Fe_3_O_4_/g-C_3_N_4_/MoO_3_ (30%);(f) HRTEM images of the Fe_3_O_4_/g-C_3_N_4_/MoO_3_ (30%) nanocomposite.

Fig 3 presented the morphology and microstructure of the MoO_3_, g-C_3_N_4_, Fe_3_O_4_/g-C_3_N_4_, and Fe_3_O_4_/g-C_3_N_4_/MoO_3_ (30%) samples investigated by TEM. It was obviously that MoO_3_ possessed Flake-like structure with the size of about 200 nm (Fig 3(A)). Pure g-C_3_N_4_ (Fig 3(B)) shows lamellar-like and smooth morphology. In Fe_3_O_4_/g-C_3_N_4_ composites (Fig 3(C)), dark Fe_3_O_4_ nanoparticles with a particle size of 10–20 nm were deposited on the surface. For Fe_3_O_4_/g-C_3_N_4_/MoO_3_ (30%) composites (Fig 3(D)), the composites of Fe_3_O_4_/g-C_3_N_4_ were well adhered on the surface of MoO_3_. These results demonstrated the successful synthesis of the ternary Fe_3_O_4_/g-C_3_N_4_/MoO_3_. To further verify the formation of Fe_3_O_4_/g-C_3_N_4_/MoO_3_ ternary structure, HRTEM image it has been used to investigate the microstructure of 30% Fe_3_O_4_/g-C_3_N_4_/MoO_3_ (Fig 3(E)). The HRTEM image illustrated that the heterostructure of Fe_3_O_4_/ g-C_3_N_4_/ MoO_3_ composite material showed lattice fringes of 0.38 nm corresponded to the (110) plane of MoO_3_, the fringes of 0.29 nm assigned to the (220) plane of Fe_3_O_4_. The interaction between MoO_3_ and g-C_3_N_4_ could benefit a continuous flow of electrons between them due to the improvement of electron channelization through the interface [43], resulting in the improvement of photocatalytic efficiency.

**Fig 4:**
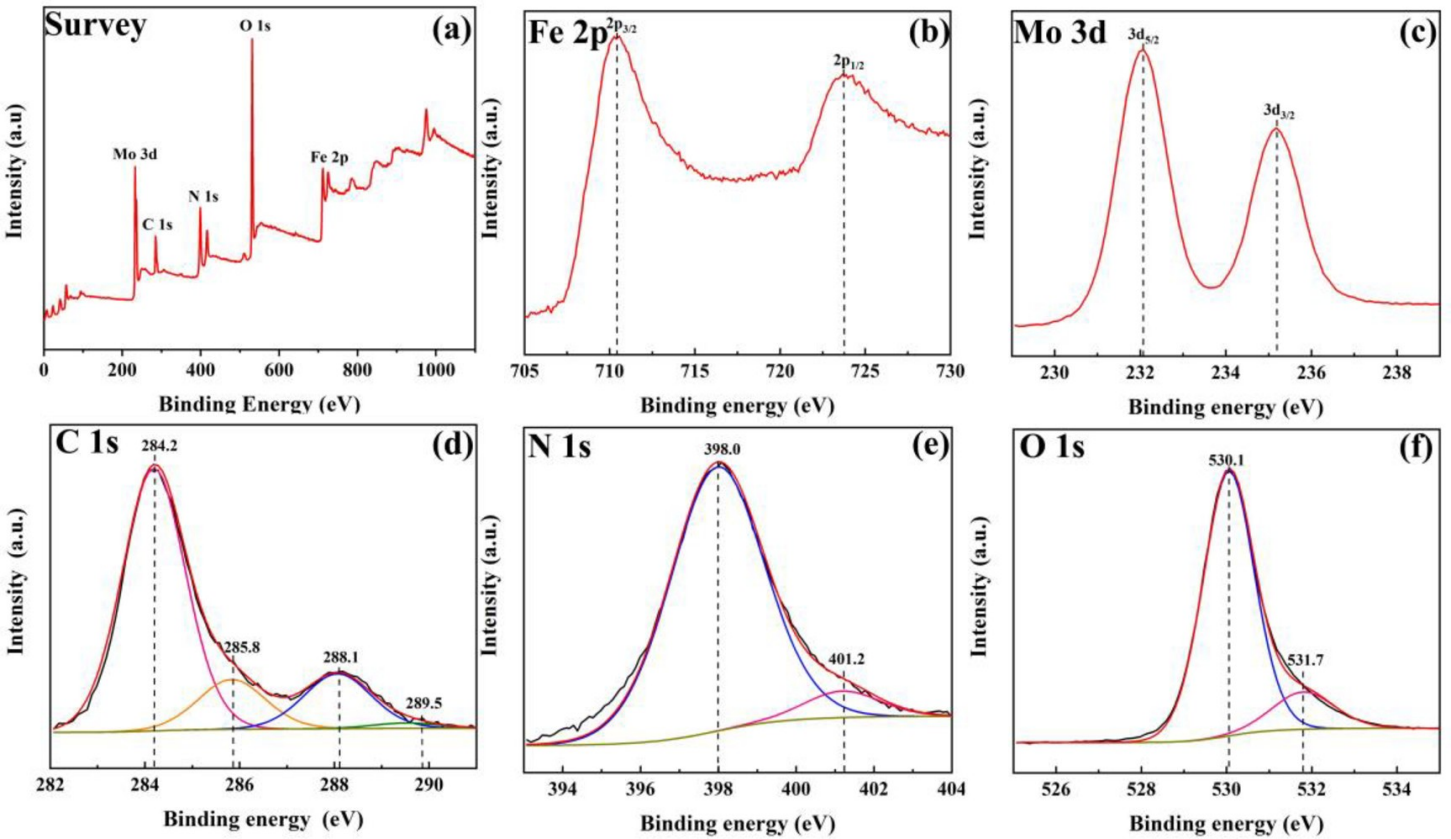
XPS patterns of Fe_3_O_4_/g-C_3_N_4_/MoO_3_ (30%) nanocomposite. (a) Survey spectra_;_ (b)Fe 2p; (c) Mo 3d; (d) C 1s; (e) N 1s; (f) O 1s.

X-ray photoelectron spectroscopy (XPS) was carried out to further analyze the surface compositions and chemical states of Fe_3_O_4_/g-C_3_N_4_/MoO_3_ (30%) sample (Fig 4). Fig 4(A) revealed the presence of Mo, C, N, O and Fe elements on the surface of the as-prepared sample. The photoelectron lines at binding energy of 233, 285, 363, 399, 531 and 712 eV were correspond to Mo 3d, C 1s, N 1s, O 1s and Fe 2p in the sample, respectively [44]. Fig 4(B) represented the XPS spectrum of Fe. The two Fe 2p_3/2_ and 2p_1/2_ peaks corresponding to binding energy 710.6 and 723.7 eV without shakeup satellite peak of Fe_2_O_3_, and their binding energy was consistent with that in pure Fe_3_O_4_ [45], suggesting the coexistence of dual iron oxidation states of Fe^2+^ and Fe^3+^ [46]. The binding energy spectrum of Mo was demonstrated in Fig 4(C), there were only two peaks existed at 232.1 and 235.3 eV corresponding to 3d_5/2_ and 3d_3/2_ of Mo atom in +6 oxidation states [47]. The C 1s signal could be divided into four peaks at 284.2, 285.8, 288.1, and 289.5 eV, implying the presence of chemically different carbon species in the sample (Fig 4(D)). The peaks located at 284.2 and 285.8 were attributed to C = C and C-O bonds, respectively [48]. The peak located at 288.1 eV was attributed to sp^2^ hybridised C atoms in the triazine rings inside thearomatic structure, while the peak at 289.5 eV was corresponded to N = C-N group or -NH_2_ group as originating from g-C_3_N_4_. The XPS peak of N 1s (Fig 4(E)) obviously centered at the binding energy of 398.0 eV, which could be assigned to the sp^2^ hybridized nitrogen (C = N-C) whereas peak at and 401.2 eV represented the tertiary nitrogen (N-C_3_). Based on Fig 4(F), there were two types of oxygen species, which should assign to the O 1s peak. The offering of the anionic oxygen in Fe_3_O_4_ centered at about 530.1 eV, and the oxygen in MoO_3_ centered at 531.7 eV [49]. The XPS results strongly suggested the coexistence of Fe_3_O_4_, g-C_3_N_4_, and MoO_3_.

**Fig 5:**
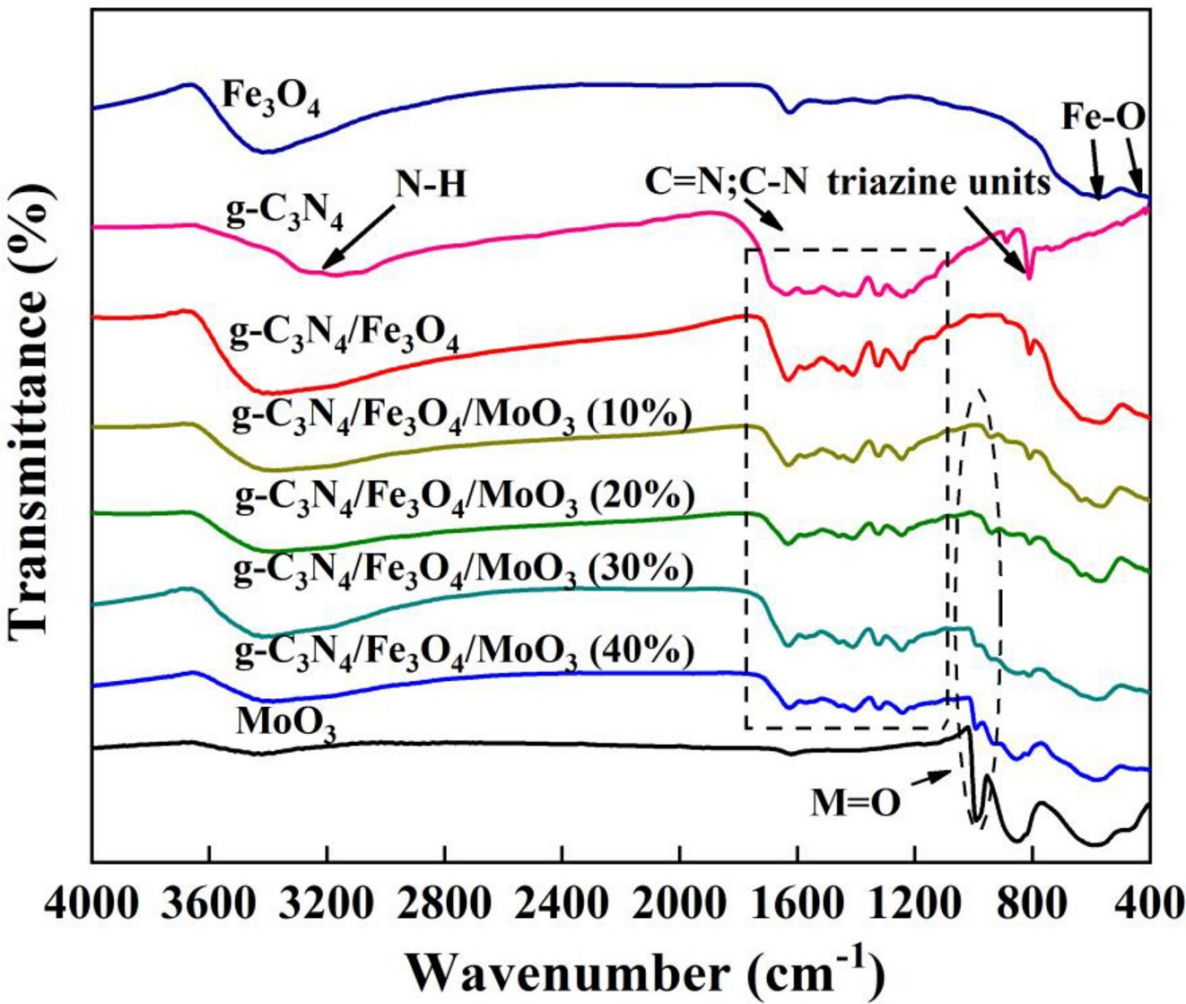
FT-IR spectra of MoO_3_, g-C_3_N_4_, Fe_3_O_4_, Fe_3_O_4_/g-C_3_N_4_ and Fe_3_O_4_/g-C_3_N_4_/MoO_3_ nanocomposites.

Chemical structures of the MoO_3_, g-C_3_N_4_, Fe_3_O_4_, Fe_3_O_4_/g-C_3_N_4_ and various Fe_3_O_4_/g-C_3_N_4_/MoO_3_ nanocomposites were studied by FT-IR spectra, and the results were exhibited in Fig 5. For pure g-C_3_N_4_, the absorption signal of 3165 cm^−1^ was beneficial from the stretching vibrations of N-H. The strong absorption band in the range of 1240–1650 cm^−1^ is correspond to typical skeletal stretching vibrations of C-N and C = N in s-triazine or tri-striazine [50]. Simultaneously, the band at 809 cm^−1^ can be assigned to the typical breathing mode of the heptazine arrangement [51]. In case of pure Fe_3_O_4_ nanoparticles, two peaks at 566 and 421 cm^−1^ were corresponded to the stretching vibrations of Fe-O [52]. Neat MoO_3_ showed signals of 599 cm^−1^ and 852 cm^−1^ which were related to the stretching vibrational modes of O shared by three Mo and the Mo-O-Mo unit respectively in the crystalline α-MoO_3_. In addition, a signal at 991 cm^−1^ was due to Mo = O for the crystalline α-MoO_3_ [53]. In the Fe_3_O_4_/g-C_3_N_4_/MoO_3_ nanocomposites, the existence of the typical vibrational modes of g-C_3_N_4_, Fe_3_O_4_, and MoO_3_ indicated the coexistence of these three contents in the nanocomposites.

S2 Fig displays TGA curves for the g-C_3_N_4_ and Fe_3_O_4_/g-C_3_N_4_/MoO_3_ (30%) samples. As can be seen, the pristine g-C_3_N_4_ shows a weight loss of about 96% after heating up to 750 ^o^C. Hence, it was concluded that the g-C_3_N_4_ decomposes almost completely heating up to 750 ^o^C. It is evident that the thermal behavior of Fe_3_O_4_/g-C_3_N_4_ and Fe_3_O_4_/g-C_3_N_4_/MoO_3_ (30%) samples are similar to that of g-C_3_N_4_. As can be seen, by loading Fe_3_O_4_ and MoO_3_ on the g-C_3_N_4_ sheets, thermal degradation of the nano-composites starts from lower temperature relative to the pristine g-C_3_N_4_. Hence, similar to many g-C_3_N_4_-based nanocomposites, thermal stability of the pristine g-C_3_N_4_ decreases with depositing different particles [45,46]. The g-C_3_N_4_ contents of Fe_3_O_4_/g-C_3_N_4_ and Fe_3_O_4_/g-C_3_N_4_/MoO_3_ (30%) nanocomposites were calculated from the weights remaining after heating the samples to over 650 ^o^C. The g-C_3_N_4_ contents of the Fe_3_O_4_/g-C_3_N_4_/MoO_3_ (30%) nanocomposite was about 8.2%, respectively. As can be seen, besides the weight loss of g-C_3_N_4_, another weight loss between 750 and 1000 ^o^C in the Fe_3_O_4_/g-C_3_N_4_/MoO_3_ (30%) composites, could be ascribed to the vaporization of MoO_3_. The MoO_3_ contents of the Fe_3_O_4_/g-C_3_N_4_/MoO_3_ (30%) is about 16.4%. In addition, after calculation, The MoO_3_ contents of the Fe_3_O_4_/g-C_3_N_4_/MoO_3_ (30%) is about 75.4%. The results were listed in Table 1.

**Table 1:** Weight percentages of different compounds in the Fe_3_O_4_/g-C_3_N_4_/MoO_3_ 30%) nanocomposite.

Compound	Weight percentage
g-C_3_N_4_	8.2
MoO_3_	16.4
Fe_3_O_4_	75.4

**Fig 6:**
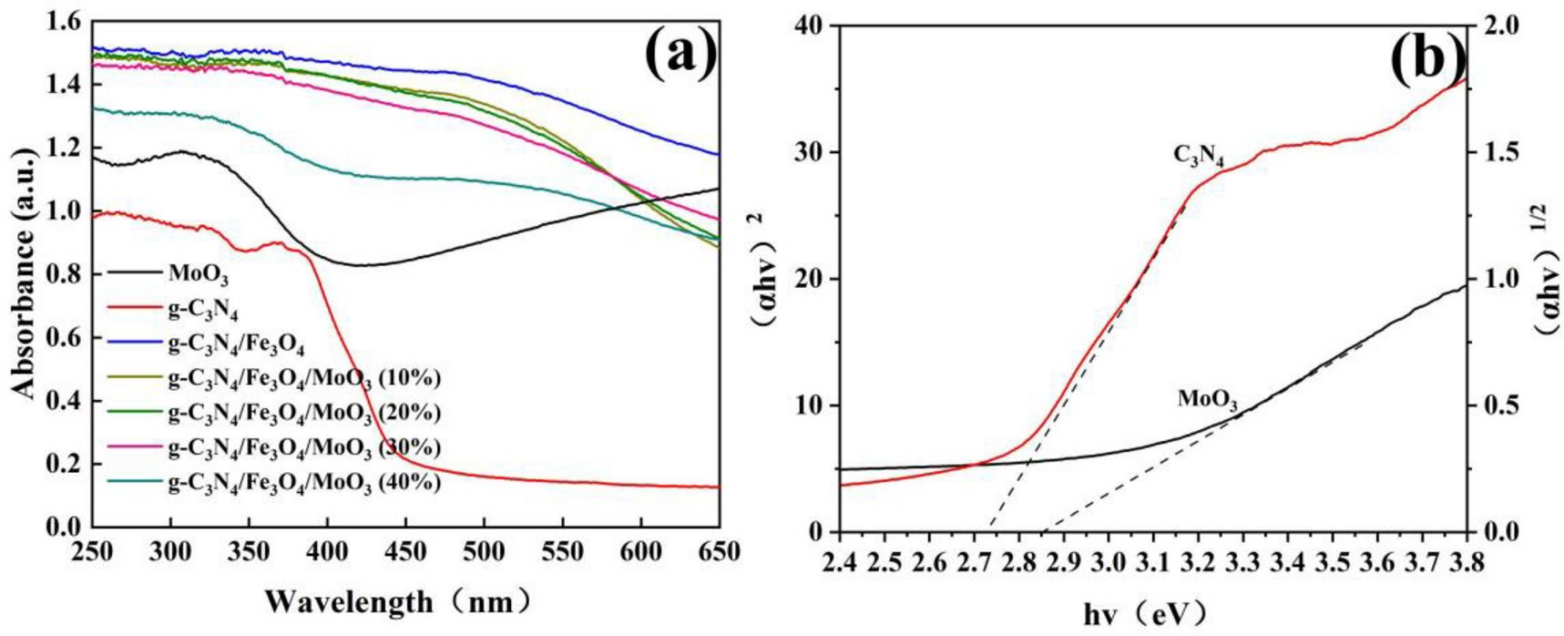
(a) UV-Vis diffuse reflectance absorption spectra for the MoO_3_, g-C_3_N_4_, Fe_3_O_4_, Fe_3_O_4_/g-C_3_N_4_ and Fe_3_O_4_/g-C_3_N_4_/MoO_3_ nanocomposites_;_ (b) The corresponding calculated band gaps of g-C_3_N_4_ and MoO_3_.

It was well known that the photoabsorptive capacity of a photocatalyst would greatly affect its photocatalytic activity [54]. Thus, UV-Vis DRS was used to investigate the photoabsorption ability of a series of as-prepared samples and the results were showed in Fig 6. As could be seen in Fig 6(A), both pristine g-C_3_N_4_ and MoO_3_ possessed small absorption in visible region and had absorption edges at about 470 nm, which were compatible with the reported absorption edges for g-C_3_N_4_ and MoO_3_ [55]. Fig 6(B) displayed the band gaps of g-C_3_N_4_ and MoO_3_ were consistent with previous studies, which were 2.72 eV and 2.85 eV, respectively [20]. The band gap of all as-prepared photocatalysts were obtained by using Taucʼs equation (Eq 1).

$$\alpha h\nu =A{\left(hv-Eg\right)}^{n/2}$$ (1)

where, *α*, *h*, *ν*, and A were absorption coefficient, Planck's constant (eV. s), the light frequency (s^−1^), and proportionality constant, respectively. *Eg* was the band gap, and *n* was the power which was assumed to be 1 and 4 for direct and indirect transitions, respectively [56,57]. As displayed in the figure, the addition of Fe_3_O_4_ to the pure g-C_3_N_4_ greatly enhanced the absorption in the visible range. Interesting, the addition of MoO_3_ to the Fe_3_O_4_/g-C_3_N_4_ slightly decreased the visible light absorption when the weight percentages of MoO_3_ were lower than 30%. The absorption would be significantly reduced when the content of MoO_3_ was over the value. However, compared to pristine g-C_3_N_4_ and MoO_3_, the visible light absorption of Fe_3_O_4_/g-C_3_N_4_/MoO_3_ nanocomposites was considerably high. These facts possibly make Fe_3_O_4_/g-C_3_N_4_/MoO_3_ to use more visible light, and produce more photoexcited charge carriers than pure g-C_3_N_4_ or MoO_3_.

**Fig 7:**
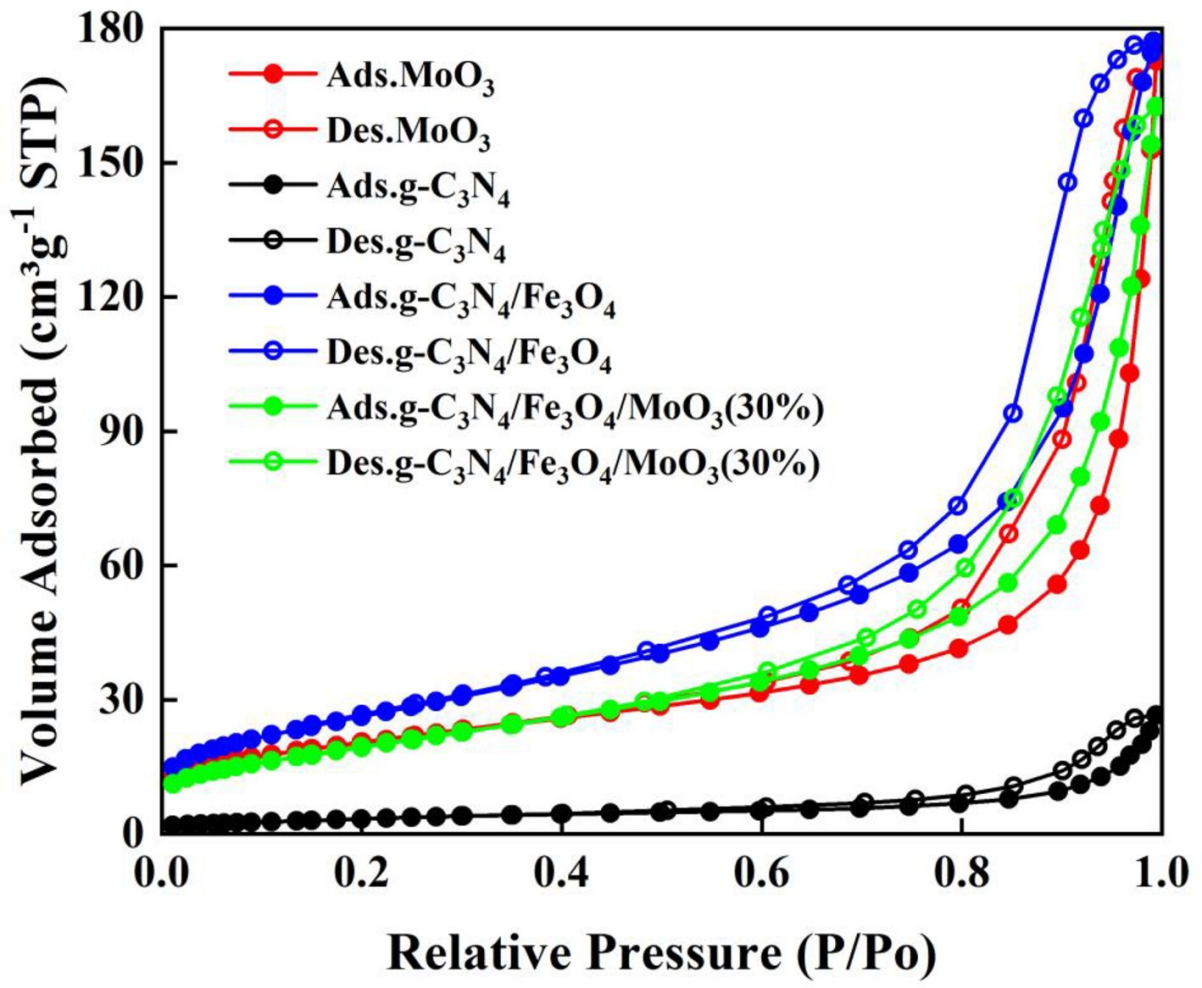
Nitrogen adsorption-desorption isotherms of MoO_3_, g-C_3_N_4_, Fe_3_O_4_, Fe_3_O_4_/g-C_3_N_4_ and Fe_3_O_4_/g-C_3_N_4_/MoO_3_ (30%) nanocomposite.

To analyze textural properties of the prepared MoO_3_, g-C_3_N_4_, Fe_3_O_4_/g-C_3_N_4_, and Fe_3_O_4_/g-C_3_N_4_/MoO_3_ (30%) photocatalysts, the results about N_2_ adsorption-desorption isotherm were provided in Fig 7. As could be seen, the isotherm of each sample was of typical IV with H_3_ hysteresis, indicating a characteristic of mesopores structure [58], which benefited to decreasing mass transfer limitations and harvesting light in the photocatalytic process [59]. BET and BJH models were used to investigate the specific surface areas and pore features of the four photocatalysts, respectively and the results were presented in Table 2. The surface areas of the MoO_3_, g-C_3_N_4_, Fe_3_O_4_/g-C_3_N_4_, and Fe_3_O_4_/g-C_3_N_4_/MoO_3_ (30%) were 73.1, 12.6, 97.4, and 72.7 m^2^g^−1^, respectively. Compared to single-phase g-C_3_N_4_, Fe_3_O_4_/g-C_3_N_4_ had larger surface area, which might attribute to the formation of hierarchical structure after loading Fe_3_O_4_ on g-C_3_N_4_ [60]. However, after the Fe_3_O_4_/g-C_3_N_4_ being modifying with MoO_3_, the surface area was decreased. This decrease probably caused by the covering of the Fe_3_O_4_/g-C_3_N_4_ surface by MoO_3_, resulting in the blocking of some active sites on the surface [61]. Generally, a decreased in the specific surface area of a semiconductor was accompanied by a decrease in its photocatalytic activity. Hence, the highly improved photocatalytic activity of Fe_3_O_4_/g-C_3_N_4_/MoO_3_ (30%) should be not described to its textural properties.

**Table 2:** The textural properties of g-C_3_N_4_, Fe_3_O_4_/g-C_3_N_4_, Fe_3_O_4_/g-C_3_N_4_/MoO_3_ (30%) samples.

Photocatalyst	Surface area (m^2^ g^-1^)	Mean pore diameter (nm)	Total pore volume (cm^3^ g^-1^)
MoO_3_	73.0615	14.63057	0.267233
g-C_3_N_4_	12.6271	13.03849	0.041159
Fe_3_O_4_/g-C_3_N_4_	97.4179	11.24809	0.273941
Fe_3_O_4_/g-C_3_N_4_/MoO_3_ (30%)	72.6855	13.84626	0.251606

**Fig 8:**
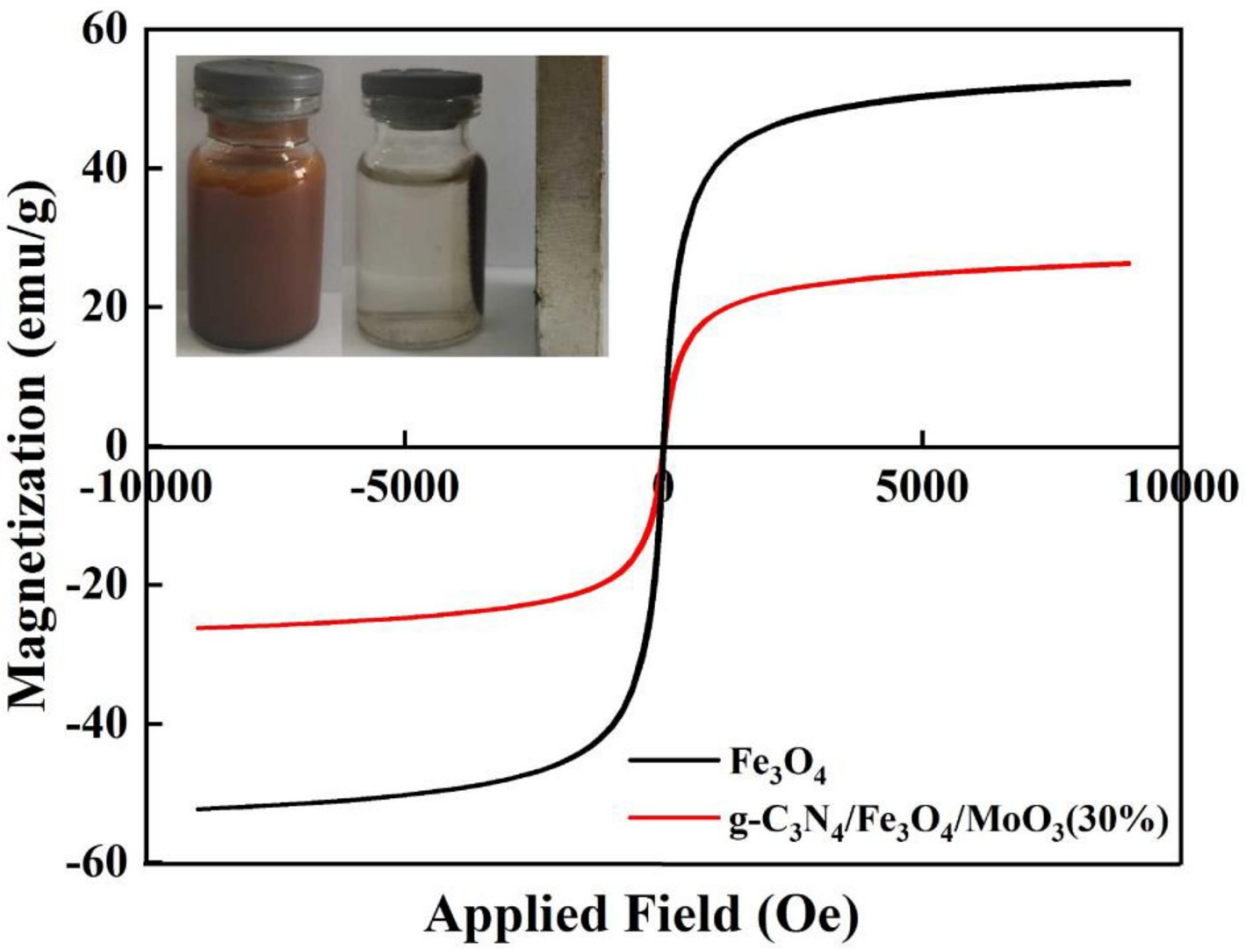
Magnetization curves for the Fe_3_O_4_ nanoparticles and Fe_3_O_4_/g-C_3_N_4_/MoO_3_ (30%) nanocomposite. Inset of the figure shows separation of the nanocomposite from the treated solution using a magnet.

Fig 8 displayed the VSM curves for the Fe_3_O_4_ nanoparticles and Fe_3_O_4_/g-C_3_N_4_/MoO_3_ (30%) photocatalyst at ambient temperature. Saturation magnetization of the Fe_3_O_4_ nanoparticles was 52.5 emu/g, while that of the Fe_3_O_4_/g-C_3_N_4_/MoO_3_ (30%) nanocomposites decreased to 26.3 emu/g due to the existence of the non-magnetic g-C_3_N_4_ and MoO_3_. However, both of the samples displayed super paramagnetic behavior. By pacing an external magnet beside the glass bottle containing the Fe_3_O_4_/g-C_3_N_4_/MoO_3_ (30%) nanocomposite, the particles were rapidly attracted to the wall of the glass bottle, as shown in the top-left inset of Fig 8, suggesting an easy separation under external magnetic field.

### 2.2. Photocatalytic activity and mechanism

**Fig 9:**
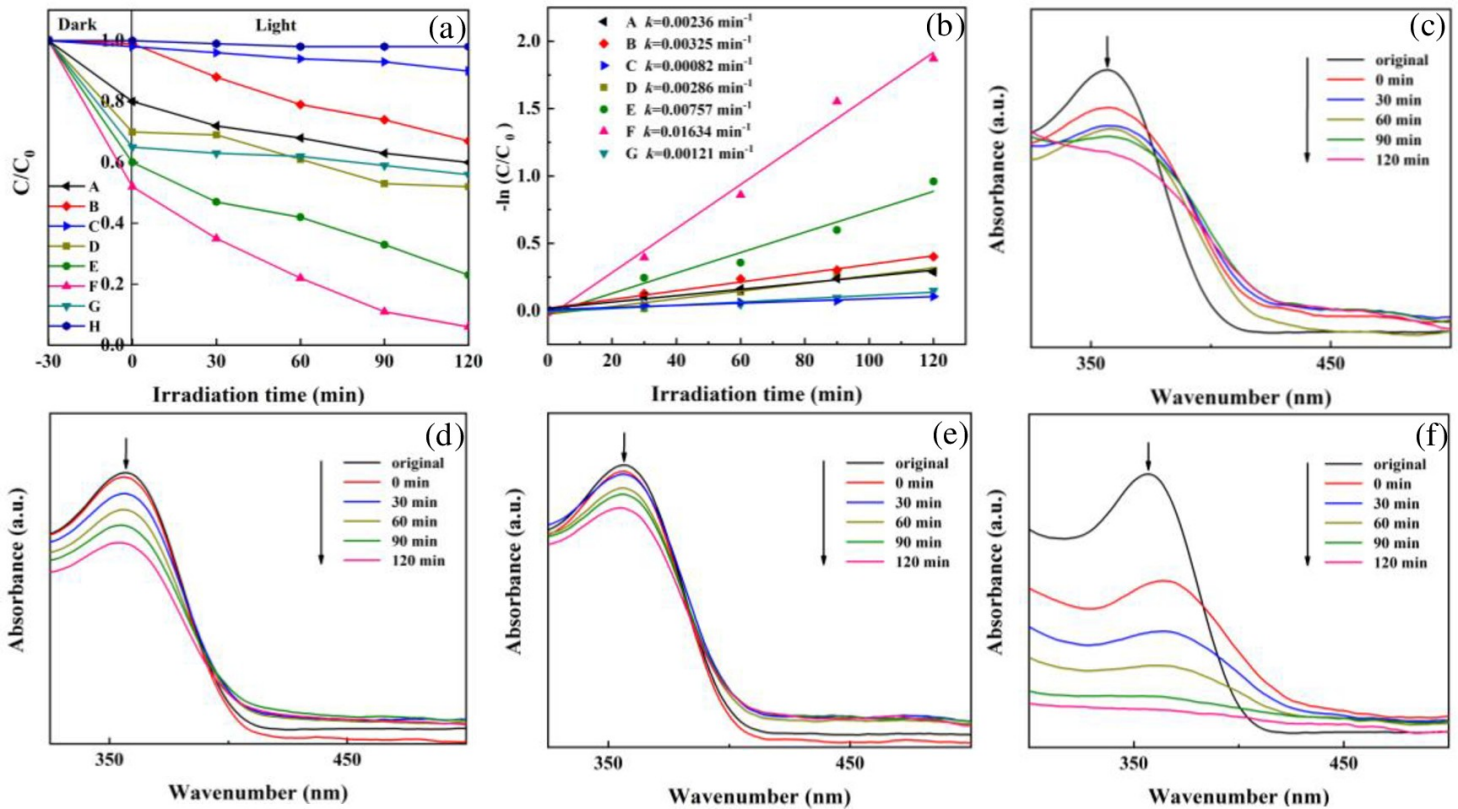
(a) Comparison of the photocatalytic activities of different samples. (A) MoO_3_; (B) g-C_3_N_4_; (C) Fe_3_O_4_/g-C_3_N_4_; (D) Fe_3_O_4_/g-C_3_N_4_/MoO_3_ (10%); (E) Fe_3_O_4_/g-C_3_N_4_/MoO_3_ (20%); (F) Fe_3_O_4_/g-C_3_N_4_/MoO_3_ (30%); (G)Fe_3_O_4_/g-C_3_N_4_/MoO_3_ (40%) (H)No photocatalyst; (b) Pseudo-first-order kinetic curves of the corresponding samples; (c), (d), (e),and (f) Temporal evolutions of the spectra during the photocatalytic degradation of TC over MoO_3_, g-C_3_N_4_, Fe_3_O_4_/g-C_3_N_4_,and Fe_3_O_4_/g-C_3_N_4_/MoO_3_ (30%).

Degradation of TC solution under visible light to evaluate the photocatalytic activity of the as-prepared catalyst, and the results were demonstrated in Fig 9. As shown in Fig 9(A), the blank experiments (in absence of any photocatalyst) revealed that the changes of TC concentration were negligible, that mean TC was quite stable under light irradiation, thus the self-degradation of TC was ruled out. The removal percentage of TC was denoted as *C/C*_*0*_, in which *C* was the TC concentration after adsorption and light illumination for a certain time, and *C*_*0*_ was the initial concentration of TC. For pristine MoO_3_, there were 20% TC were adsorbed and only about 17% TC were photodegraded in 120 min. Single-phase g-C_3_N_4_ displayed almost no adsorption and moderate photocatalytic activity for TC, with a removal percentage of 28% after 120 min under visible light. It should be noted that, when the Fe_3_O_4_ loaded on g-C_3_N_4_, the photodegrading ability decreased, with a removal percentage of 10%, implying Fe_3_O_4_ had a negative effect on the photocatalytic activities. The incorporation of the MoO_3_ boosted the overall activity, and the Fe_3_O_4_/g-C_3_N_4_/MoO_3_ composites displayed remarkable enhancements in the photodegrading-abilities. After irradiated for 120 min, the removal percentages were about 48, 77, 94, and 44% for 10, 20, 30, and 40% Fe_3_O_4_/g-C_3_N_4_/MoO_3_ nanocomposites, in which 18, 37, 46, and 9% were attributed to photodegradation, respectively. Obiviously, the nanocomposites with 30% MoO_3_ possessed the best photocatalytic activity. Since the Fe_3_O_4_ had no positive effect on the photodegrading-ability, the improvements in the photacatalytic performance of the nanocomposites should attribute to the cooperation of g-C_3_N_4_ and MoO_3_. Furthermore, when the weight percent of MoO_3_ was over 30%, the degradation of TC decreased. That was to say, excess load of MoO_3_ leaded to the lower photodegradating-ability, which implied that the superfluous MoO_3_ could impede the interaction of g-C_3_N_4_ and MoO_3_. The pseudo-first-order kinetic model (ln[*TC*] = ln[*TC*]_0_−*k_obs_t*) was used to fit with the degradation process to quantify the activities of the resultant samples, in which the value of the observed first-order rate constant (*k*_*obs*_) was equal to the corresponding slope of the straight line [62]. As shown in Fig 9(B), The *k* of MoO_3_, g-C_3_N_4_, Fe_3_O_4_/g-C_3_N_4_, and Fe_3_O_4_/g-C_3_N_4_/MoO_3_ (30%) nanocompositeis were 2.36×10^−3^, 3.25×10^−3^, 8.2×10^−4^, and 1.63×10^−2^ min^−1^, respectively. Thus, it could be concluded that activity of the Fe_3_O_4_/g-C_3_N_4_/MoO_3_ (30%) nanocompositeis was about 6.9, 5 and 19.9-fold higher than those of MoO_3_, g-C_3_N_4_, and Fe_3_O_4_/g-C_3_N_4_ composites, respectively. Fig 9(C)–9(F) displayed the UV-Vis spectral variation of TC solution during the adsorption and photodegradation over the MoO_3_, g-C_3_N_4_, Fe_3_O_4_/g-C_3_N_4_, and Fe_3_O_4_/g-C_3_N_4_/MoO_3_ (30%) nanocompositeis. For all the samples, the maximal absorbance at 355 nm decreased as the reaction progressed, suggesting gradual removal of TC. Comparison of Fe_3_O_4_/g-C_3_N_4_/MoO_3_ (30%) with other similar reported systems of Fe_3_O_4_/g-C_3_N_4_ composites has been discussed in S1 Table.

**Fig 10:**
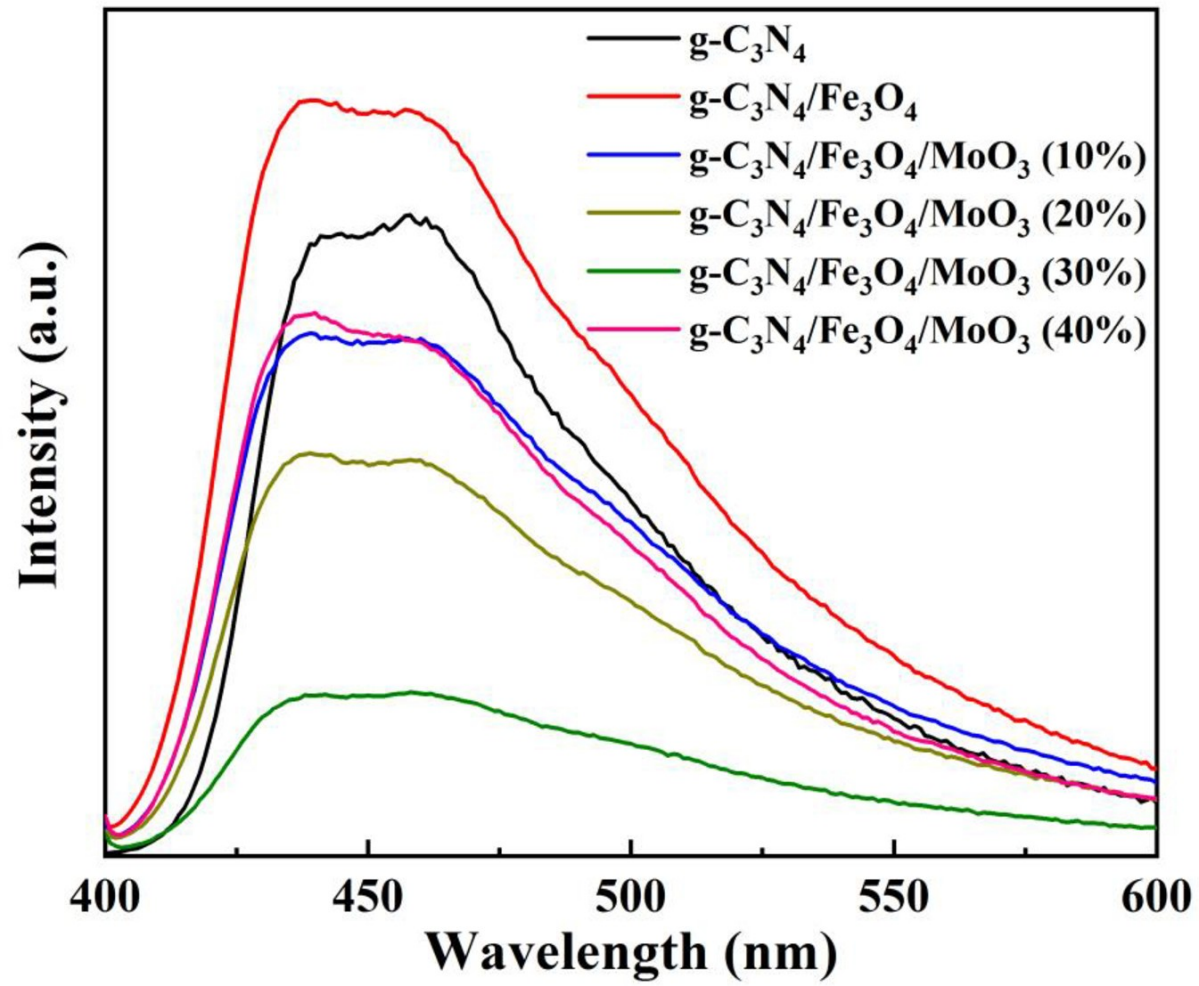
PL spectra of g-C_3_N_4_, Fe_3_O_4_, Fe_3_O_4_/g-C_3_N_4_ and Fe_3_O_4_/g-C_3_N_4_/MoO_3_ nanocomposites.

In generally, for most semiconductors the photo-induced e^−^/h^+^ pairs can recombine after being irradiated by light thus emit fluorescence, which can be indicated by PL. Higher PL intensity of a semiconductor indicates a higher recombination rate of its e^−^/h^+^ pair [63]. Fig 10, showed the PL spectroscopy of g-C_3_N_4_, Fe_3_O_4_/g-C_3_N_4_, and Fe_3_O_4_/g-C_3_N_4_/MoO_3_ series samples. As seen in the figure, g-C_3_N_4_ displayed large PL signal due to the high recombination of photo-induced e^−^/h^+^ pairs and low quantum yield [16]. However, Fe_3_O_4_/g-C_3_N_4_ nanocomposites exhibited a stronger PL than that of the pure g-C_3_N_4_, indicating a lower separation rate of photo-induced e^−^/h^+^ pairs. Interestingly, the addition of MoO_3_ to the Fe_3_O_4_/g-C_3_N_4_ nanocomposites followed by the formation of the Fe_3_O_4_/g-C_3_N_4_/MoO_3_ obviously reduced the PL emission intensity due to the combination of MoO_3_ and Fe_3_O_4_/g-C_3_N_4_, which suggested the fabrication of the nanocomposites efficiently enhanced the of separation of e^−^/h^+^ pairs on the surface. It should be noted that the PL signal increased significantly when the content of MoO_3_ were over 30%, implying an easier recombination of photogenerated charge carriers. The incensement may attribute to the agglomeration of the overloaded MoO_3_ on the surface of the nanocomposites, resulting in the reduction of the interface area between g-C_3_N_4_ and MoO_3_.

**Fig 11:**
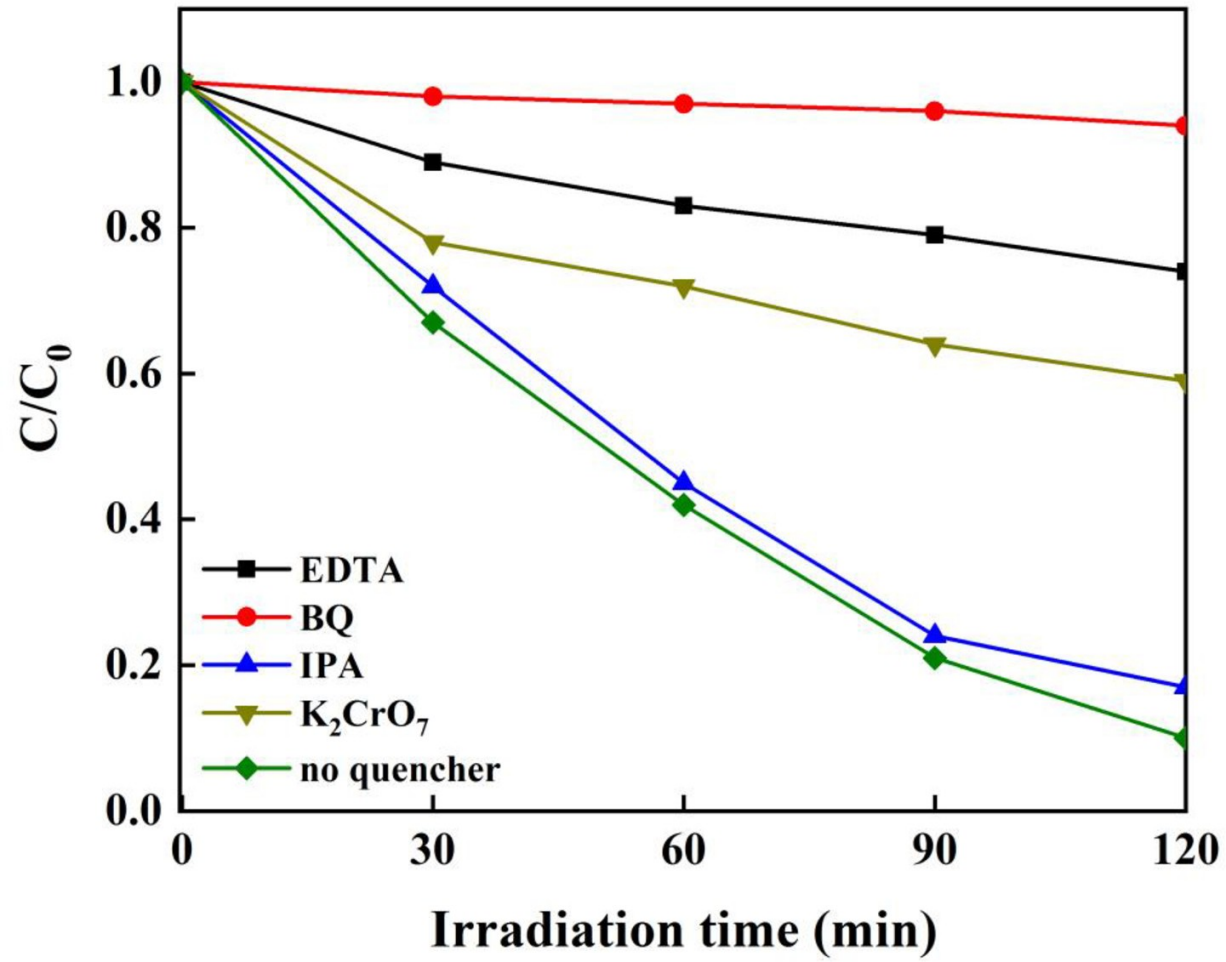
Results of active species trapping experiments.

It had been reported that the ·O_2_^−^, ·OH, e^−^ and h^+^ were the main active species attributed to the photodegradation contaminants during the photocatalytic reactions [64]. However, their contribution to the degradation of contaminants was not identical and could be investigated by utilizing the quenching experiments. In order to estimate the role of each radical in the TC photodegradation, EDTA-2Na, K_2_Cr_2_O_7_, IPA, and BQ were respectively used as the quenchers for h^+^, e^−^, ·OH, and ·O_2_^−^ in the TC degradation process in the Fe_3_O_4_/g-C_3_N_4_/MoO_3_ (30%) system. As shown in Fig 11, the degradation percentage of TC after 120 min irradiation was 90% with free quencher and drastically decreased to about 6% when BQ was added into the system. In the same time, the addition of EDTA-2Na, K_2_Cr_2_O_7_ and IPA resulted in 21, 39 and 83% photodegadation percentages of TC. These results indicated that ·O_2_^−^ played vital role for the TC photodegradation, h^+^ and e^−^ had modest contribution to the TC decomposition, while ·OH was had the lowest contribution to the TC degradation. It should be noted that some other intermediates might be produced during the photodegradation reaction, which might take part in the degradation of TC.

In composites of two semiconductors, the effective separation of e^−^/h^+^ pairs depends on the appropriate band-gap positions of them. The band positions of g-C_3_N_4_ and MoO_3_ could be obtained using empirical equations (Eqs 2 and 3) [23]:

$$E_{CB}=X-E_{C}-\frac{1}{2}E_{g}$$ (2)

$$E_{VB}=E_{CB}+E_{g}$$ (3)

Where *X* is the absolute electronegativity of the atom semiconductor used to represent the geometric mean of the absolute electro-negativity of the constituent atoms, which is defined as the arithmetic mean of the atomic electron affinity and the first ionization energy; *E*_*CB*_ is the energy of free electrons of the hydrogenscale (4.5 eV); *E*_*g*_ is the band gap of the semiconductor; E_CB_ is the conduction band potential and *E*_*VB*_ is the valence band potential. According to previous studies, the absolute electronegativity *X* for g-C_3_N_4_ and MoO_3_ were 4.73 eV and 6.40 eV [65,66], respectively. From the Taucʼs equation, *E*_*g*_ of g-C_3_N_4_ and MoO_3_ were to be 2.72 eV and 2.85 eV, respectively.

**Fig 12:**
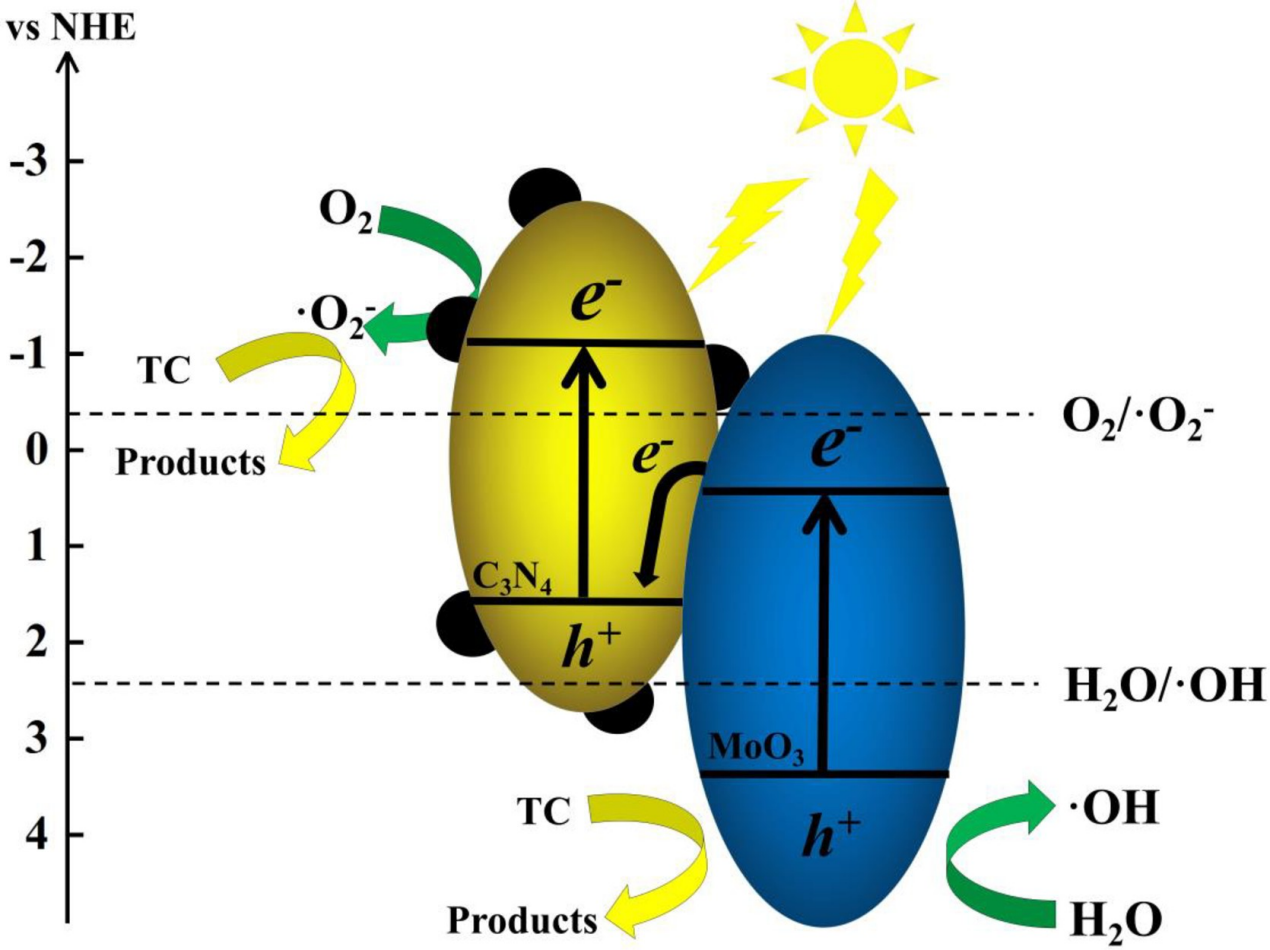
Proposed mechanism for enhanced photocatalytic activity over Fe_3_O_4_/g-C_3_N_4_/MoO_3_ nanocomposites.

Based on the above analysis, the conduction bands (CB) of g-C_3_N_4_ and MoO_3_ respectively were -1.13 and 0.47. Accordingly, the valance bands (VB) of them were 1.59 and 3.33, respectively. The results were similar to other studies [23]. Based on the results obtained by PL experiments, for the Fe_3_O_4_/g-C_3_N_4_/MoO_3_ (30%) photocatalyst, the photogenerated e^−^ and h^+^ could be effectively separated under visible light. According to the traditional mechanism, the e^−^ in the CB of g-C_3_N_4_ could transfer to the CB of MoO_3_ while the h^+^ could migrate in the opposite direction. Generally, the reduction of O_2_ with photoelectrons produced ·O_2_^−^ (e^−^+ O_2_→ ·O_2_^−^, O_2_/·O_2_^−^ = -0.33 V vs. NHE) [67]. The ·OH^−^ could be obtained by photoholes oxidized H_2_O directly (h^+^ + H_2_O → ·OH + H^+^, ·OH/OH^−^ = 2.40 V vs. NHE) (Michael R. Hoffmann, 1995; Wen et al., 2017) or indirectly through ·O_2_^−^ (·O_2_^−^+ H_2_O → H_2_O_2_ →·OH) [68]. In summary, the VB of MoO_3_ and g-C_3_N_4_ are excited by visible light at the same time, and then the photoelectrons in the CB of MoO_3_ and the holes in the solid-solid contact interface of the VB of g-C_3_N_4_ recombine, resulting in photoelectron retention In the CB of g-C_3_N_4_, holes are left in the VB of MoO_3_. Therefore, g-C_3_N_4_ and MoO_3_ could form Z-scheme and enhanced the separation of photogenetrated e^−^/h^+^ pairs at the interface of Fe_3_O_4_/g-C_3_N_4_/MoO_3_ [64]. As shown in Fig 12, under light irradiation, the e^−^ in the CB of g-C_3_N_4_ had relative stability thus benefited to the continuous generation of O_2_^−^ from O_2_. The h^+^ in the VB of MoO_3_ generating ·OH^−^ by oxidized H_2_O. Some of h^+^ in the VB of MoO_3_ took part in the oxidation of TC, while the rest h^+^ were reduce H_2_O to^.^OH, which was not the main reactive species for TC degradation.

## 4. Conclusions

In this study, a novel and easily separated ternary Fe_3_O_4_/g-C_3_N_4_/MoO_3_ (30%) photocatalyst was presented using melamine, FeCl_3_, FeCl_2_, and AHM as materials. This catalyst provided enhanced photocatalytic activity toward the removal of TC in aqueous environment. The photocatalytic activity of the novel catalyst was approximately 6.9 times of MoO_3_, 5 times of g-C_3_N_4_, and 19.9 times of Fe_3_O_4_/g-C_3_N_4_ on photodegradation of TC. The excellent photodegrading ability was due to the formation of Z-scheme structure between C_3_N_4_ and MoO_3_, which could effectively separate the photogenerated e^−^/h^+^ pairs and efficiently utilize the e^−^ and h^+^. The highly improved TC-photodegrading ability was also beneficial from the wide range light absorption. This work indicated that the novel Fe_3_O_4_/g-C_3_N_4_/MoO_3_ was beneficial in decreasing TC and other environmental pollutants with high-level concentration in water, and paved a new way to the development of photocatalytic technology.

## Supporting information

S1 Fig Schematic diagram of photocatalytic reaction device. (DOC)

S2 Fig TGA curves of pure g-C_3_N_4_ and Fe_3_O_4_/g-C_3_N_4_/MoO_3_ (30%) photocatalysts. (DOC)

S1 Table Comparison of degradation performance of similar photocatalysts. (DOC)
