# Supplementary figures and images for: Novel magnetic Fe3O4/g-C3N4/MoO3 nanocomposites with highly enhanced photocatalytic activities: Visible-light-driven degradation of tetracycline from aqueous environment
Source: PLoS One. 2020 Aug 14;15(8):e0237389. doi: 10.1371/journal.pone.0237389 (PMC7428356; doi:10.1371/journal.pone.0237389)

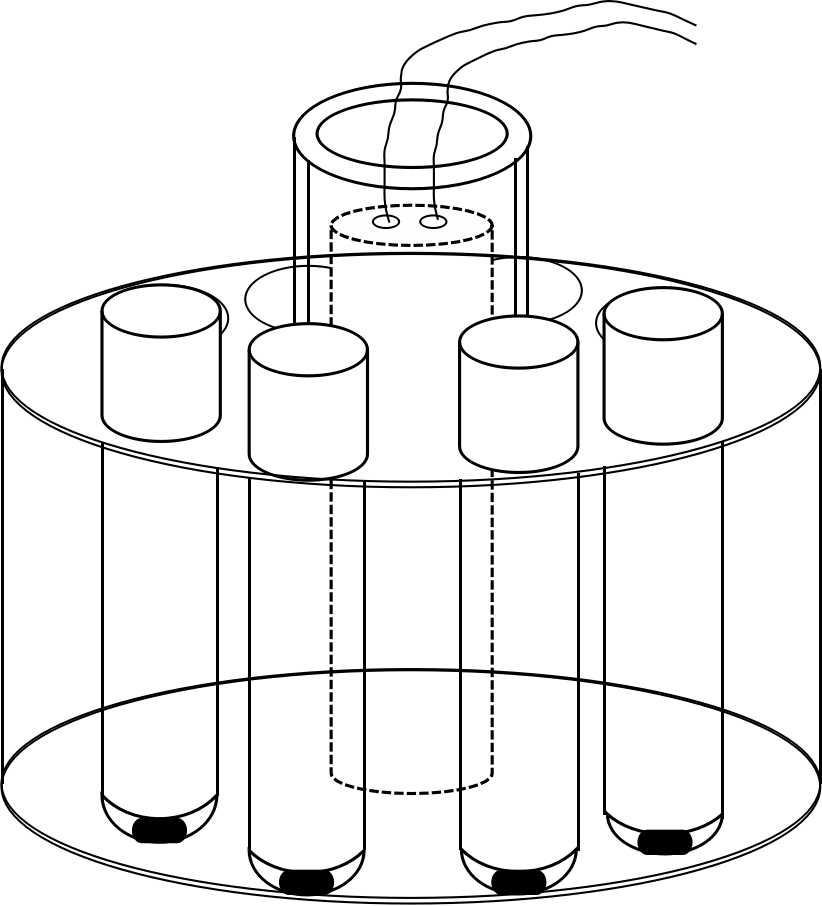


S1 Fig. Schematic diagram of photocatalytic reaction device.

Supplement: S1 Fig — (DOC) [file pone.0237389.s001.doc]

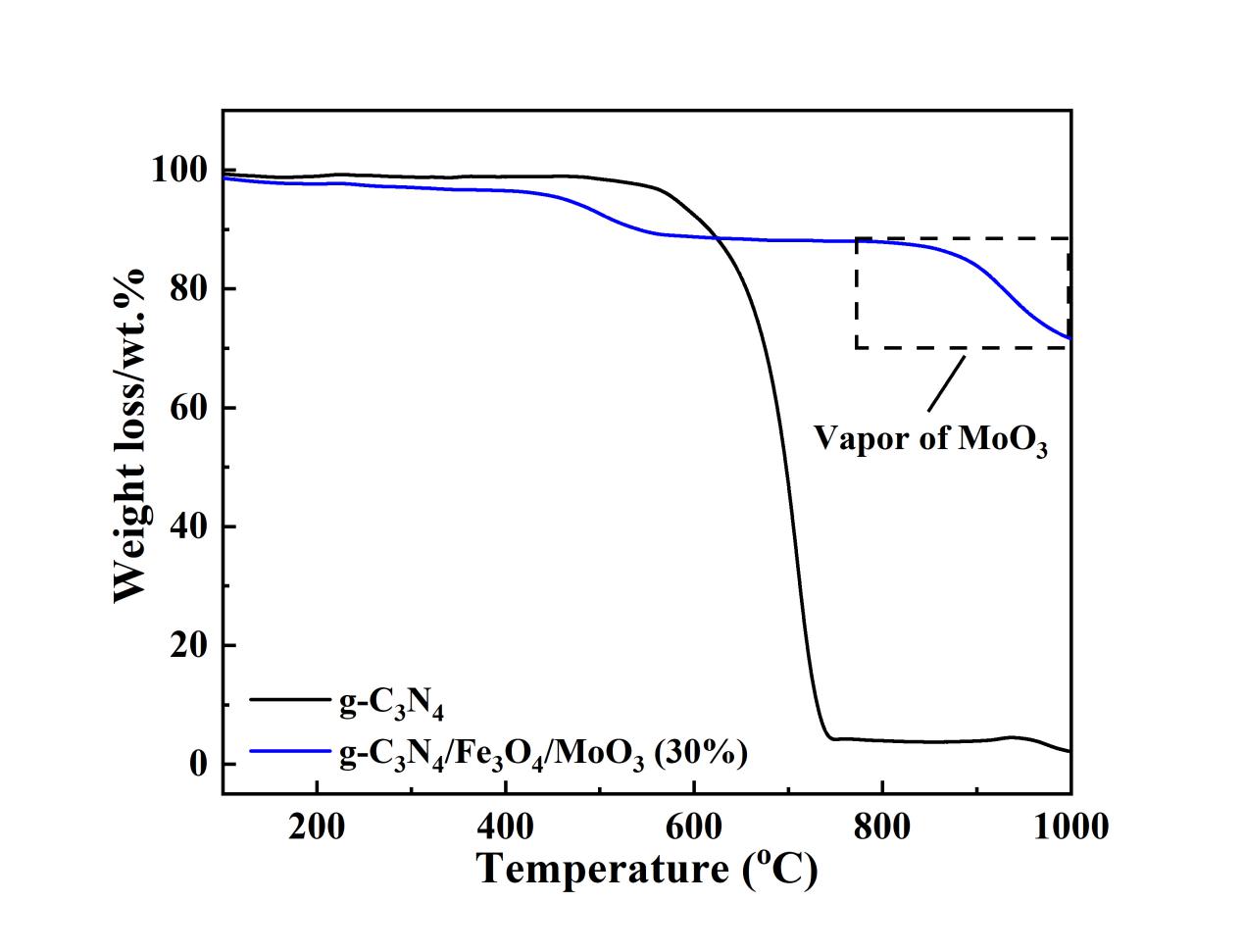


S2 Fig. TGA curves of pure g-C3N4 and Fe3O4/g-C3N4/MoO3 (30%) photocatalysts.

Supplement: S2 Fig — (DOC) [file pone.0237389.s002.doc]
