# Supplementary material for: Novel magnetic Fe3O4/g-C3N4/MoO3 nanocomposites with highly enhanced photocatalytic activities: Visible-light-driven degradation of tetracycline from aqueous environment
Source: PLoS One. 2020 Aug 14;15(8):e0237389. doi: 10.1371/journal.pone.0237389 (PMC7428356; doi:10.1371/journal.pone.0237389)
Supplement: S1 Table — (DOC) [file pone.0237389.s003.doc]

S1 Table. Comparison of degradation performance of similar photocatalysts.

| S.  No. | Photocatalysts | Source of  illumination | C_photocatlyst_  (mg mL^−1^) | C_pollutant_  (mg L^−1^) | Time  (min) | Pollutant | Photocatalytic degradation  efficiency (%) | Refs. |
| --- | --- | --- | --- | --- | --- | --- | --- | --- |
| 1 | g-C_3_N_4_/MoO_3_(7%) | 300W Xenon lamp | 1 | 10 | 180 | MB | 93 | 23 |
| 2 | 1.5 wt% MoO_3_-C_3_N_4_ | 350W Xenon lamp | 1 | 20 | 120 | MO | 87 | 30 |
| 3 | CNFO-15.2 | 300 W Xenon lamp | 0.25 | 5 | 60 | RhB | 97 | 35 |
| 4 | Fe_3_O_4_/g-C_3_N_4_/MoO_3_(30%) | 1000W  Xenon lamp | 0.2 | 40 | 120 | TC | 94 | This work |
