# Supplementary material for: Novel magnetic Fe3O4/g-C3N4/MoO3 nanocomposites with highly enhanced photocatalytic activities: Visible-light-driven degradation of tetracycline from aqueous environment
Source: PLoS One. 2020 Aug 14;15(8):e0237389. doi: 10.1371/journal.pone.0237389 (PMC7428356; doi:10.1371/journal.pone.0237389)
Supplement: Attachment — Submitted filename: reviewer comemts Fe3O4g-C3N4MoO3 nanocomposite.pdf [file pone.0237389.s004.pdf]

Manuscript Number: PONE-D-20-07918

Manuscript Title: Novel magnetic Fe_3O_4/g-C_3N_4/MoO_3 nanocomposites with highly enhanced photocatalytic activities: visible-light-driven degradation of tetracycline from aqueous environment

The article reports on the synthesis of Fe_3O_4/g-C_3N_4/MoO_3 photocatalytic material and its application towards TC degradation. There are reports on graphitic carbon nitride and it combined with other systems, and their applications in photocatalysis, sensor technology, water splitting and so on. However, this work would be a valuable addition to the field and enrich the readership. But the manuscript in its present form needs to address some critical technical points, therefore, I recommend the publication of this manuscript in Journal PLOS ONE after addressing some technical aspects, grammatical and typographical errors.

1. Update references with recent examples describing the use of visible light responding g-C_3N_4 based photocatalysts *(RSC Advances, 9 (2019), 15381-15391; Journal of Nanoscience and Nanotechnology, 19 (8) (2019), 5241-5248; Physica E: Low-dimensional Systems and Nanostructures, 114 (2019), 113560; International Journal of Hydrogen Energy, doi:10.1016/j.ijhydene.2019.06.06. ; ACS Omega 2020, 5, 8, 3828-3838)*
2. There are various typo errors throughout the manuscript, for instance in line no. 4 "," should be removed after MoO3, line no. 20 "." should be checked for pg no. 8 and similarly for whole manuscript.
3. Author has reported the effect of weight percent of MoO_3 present in the composite but no evidence has been provided to confirm the composite elaborated weight composition of final ternary composite, therefore TGA should be done to support the stated fact.
4. Author should mention the complete details of photocatalytic instrument taken in use for the degradation studies like its capacity, the distance of light source from the surface of pollutant and light source intensity etc.
5. XRD patterns graph should be expressed more clearly, the peaks of g-C3N4 in 10% and g-C3N4/Fe3O4 composite is not clearly visible in present graph style.

6. Authors must explain that how the compound weight percentage has been calculated from EDX data? EDX analysis can only give estimation for elemental composition and not compound in particular. The EDX analysis needs reconsideration.
7. Fig 2(b,C) should also be represented with corresponding elements.
8. As mentioned “Pure g-C3N4 (Fig. 3(b)) consisted of a transparent sheet structure” is not appearing as explained in TEM image. In image 3b the sheet like structure is not visible therefore need explanation or replacement.
9. The language should be improved throughout the manuscript.
