# Supplementary material for: Novel magnetic Fe3O4/g-C3N4/MoO3 nanocomposites with highly enhanced photocatalytic activities: Visible-light-driven degradation of tetracycline from aqueous environment
Source: PLoS One. 2020 Aug 14;15(8):e0237389. doi: 10.1371/journal.pone.0237389 (PMC7428356; doi:10.1371/journal.pone.0237389)
Supplement: Attachment — Submitted filename: Response to Reviewers.doc [file pone.0237389.s005.doc]

Responses to Reviewers’ Comments

Dear Editor and Reviewers:

Thank you very much for making a scrutiny into our manuscript. We appreciate editor and reviewers very much for their positive and constructive comments and suggestions on our manuscript which are all valuable and helpful for revising and improving our paper. The manuscript has been carefully revised according to the comments and suggestions, the revised parts are marked in red. Responses to the comments and suggestions are listed below. We sincerely hope the modification can conform to the request of yours. We sincerely hope this manuscript will be finally acceptable to be published on ***Plos One***. Thank you very much for all your help and looking forward to hearing from you soon.

**Reviewer #1:**

**Comment 1:**

-There are some typo errors, for example; space between two words/ two sentences. I would suggest to go through the manuscript again to remove these typo errors.

**Response:**

Thanks for your indication and suggestion.After reading the manuscript many times, these typing errors have been further corrected. We are very sorry for this negligence.

**Comment 2:**

-UV-vis word in abstract should be replaced with UV-Vis and check it throughout the manuscript.

**Response:**

Thanks for your indication and suggestion.After many inspections, We have re-checked the full text to eliminate this error, which has been corrected and marked in red. A total of three errors were found, including line 11 on page 1, line 11 on page 17, and line 7 on page 33. Sorry for this negligence.

**Comment 3:**

-The light intensity should be provided when conducting photocatalytic experiments.

**Response:**

Thank you for your kind suggestion. Based on your kind suggestions, we detected the light intensity in the photocatalytic experiment, and the result was displayed in line 1-5 page 7 in the MS, which expressed as: The capacity of the synthesized catalysts to photodegrade TC was performed by a photochemistry reaction instrument (YM-GHX-V, Shanghai Yuming Instrument Co. Ltd, China) with a 1000 W Xe lamp applied as visible light source, as shown in Fig. S1. In the reaction system, the reaction solution is packed in a quartz tube with a capacity of 50 ml, and the quartz tube is fixed at a distance of 2 cm from the light source. An optical power meter (OPT-1A, China) was used to measure the intensity of the experimental lamp to be 37.5 mW/cm^2^ (λ >400 nm). A water circulation system was utilized to keep the reaction system at 15 ^o^C. In each experiment, 10 mg of the photocatalyst was added into 50 mL of TC solution (40 mg/L).

Fig. S1 Schematic diagram of photocatalytic reaction device.

**Comment 4:**

-There are many literature available with similar kind of work and results. All the adsorbents reported be compared in a tabular form to establish the superiority of the work.

**Response:**

Thanks for your indication and suggestion. According to recommendations, We compared the photocatalytic performance of the photocatalyst in this study with some heterostructure photocatalysts in other studies to illustrate the advantages of this work. It is mentioned atline 14 page 17 in the MS and the results are listed in Table S1, which written as the following: Comparison of Fe_3_O_4_/g-C_3_N_4_/MoO_3_ (30%) with other similar reported systems of Fe_3_O_4_/g-C_3_N_4_ composites has been discussed in Table S1.

| S.  No. | Photocatalysts | Source of  illumination | C_photocatlyst_  (mg mL^−1^) | C_pollutant_  (mg L^−1^) | Time  (min) | Pollutant | Photocatalytic degradation  efficiency (%) | Refs. |
| --- | --- | --- | --- | --- | --- | --- | --- | --- |
| 1 | g-C_3_N_4_/MoO_3_(7%) | 300W Xenon lamp | 1 | 10 | 180 | MB | 93 | 23 |
| 2 | 1.5 wt% MoO_3_-C_3_N_4_ | 350W Xenon lamp | 1 | 20 | 120 | MO | 87 | 30 |
| 3 | CNFO-15.2 | 300 W Xenon lamp | 0.25 | 5 | 60 | RhB | 97 | 35 |
| 4 | Fe_3_O_4_/g-C_3_N_4_/MoO_3_(30%) | 1000W  Xenon lamp | 0.2 | 40 | 120 | TC | 94 | This work |

Tab. S1 Comparison of degradation performance of similar photocatalysts.

**Comment 5:**

-The quality of figures 2, 3 and 9 should be enhanced. However, the scale bar is not clear in figure 2, so please redraw the figure. In result and discussion ,paragraph second if fig 2 (d, e and f) is Fe,C and N then what is fig 2(b and c). Moreover fig 2 (b,c,d,e and f) is not mentioned anywhere in the manuscript.

**Response:**

Thank you for your kind suggestion. Based on your suggestions, we have redrawn figures 2, 3 and 9, and re-explained and accurately explained and analyzed Fig 2 (d, e, and f) in the 5 line of the 10 page of the manuscript, which expressed as: It could be clearly found C, N, Fe, O and Mo (Fig. 2(b-f)) were all homogeneous indicating uniform distributions of Fe_3_O_4_, g-C_3_N_4_, and MoO_3_ in the selected area of the corresponding SEM image (Fig. 2a).

**Comment 6:**

-The advantages of using composites, especially heterostructures need be introduced and the following papers need be cited: Synthesis, characterization and application of silver doped graphitic carbon nitride as photocatalyst towards visible light photocatalytic hydrogen evolution, International Journal of Hydrogen Energy(2019), Effect of Calcination Temperature, pH and Catalyst Loading on Photodegradation Efficiency of Urea Derived Graphitic Carbon Nitride towards Methylene Blue Dye Solution, Royal Society of Chemistry(2019), 9(15381-15391). ZnO-Modified g-C_3_N_4_: A Potential Photocatalyst for Environmental Application, American Chemical Society(2020). Biogenic mediated Ag/ZnO nanocomposites for photocatalytic and antibacterial activities towards disinfection of water, Journal of Colloid and Interface Science, (2020),563(370-380), Phytoextract mediated ZnO/MgO nanocomposites for photocatalytic and antibacterial activities, Journal of Photochemistry and Photobiology A: Chemistry(2019),385(112049).
**Response:**

Thanks for the reviewer’s good evaluation and kind suggestion. We read these papers carefully and found their works were helpful for ours, so we have cited them as reference as [8], [9], [13], [17], [22]. The revised section was marked as red letters in lines 6, 14, and 17 on page 3, lines 1 on page 4, which expressed as: Some photocatalysts have the function of degrading pollutants while Excellent antibacterial activity [8-9]. Due to its advantages of low toxicity, low preparation cost and high stability, it has been applied to the removal of organic pollutants in water, which has aroused extensive research interest [13-14]. Therefore, various methods have been evolved to enhance the photocatalytic activity of pure g-C_3_N_4_, including metal deposition [17-18]. By coupling g-C_3_N_4_ with other semiconductors to form a heterojunction structure, the shortcomings of high recombination rate of photogenerated electron-hole pairs of a single photocatalyst could be solved [22].

**Reviewer #2:**

**Comment 1:**

-Update references with recent examples describing the use of visible light responding g-C_3_N_4_ based photocatalysts (RSC Advances, 9 (2019), 15381-15391; Journal of Nanoscience and Nanotechnology, 19 (8) (2019), 5241-5248; Physica E: Low-dimensional Systems and Nanostructures, 114 (2019), 113560; International Journal of Hydrogen Energy, doi:10.1016/j.ijhydene.2019.06.06.; ACS Omega 2020, 5, 8, 3828-3838) Royal Society of Chemistry(2019), 9(15381-15391). ZnO-Modified g-C_3_N_4_: A Potential Photocatalyst for Environmental Application, American Chemical Society(2020). Biogenic mediated Ag/ZnO nanocomposites for photocatalytic and antibacterial activities towards disinfection of water, Journal of Colloid and Interface Science, (2020),563(370-380), Phytoextract mediated ZnO/MgO nanocomposites for photocatalytic and antibacterial activities, Journal of Photochemistry and Photobiology A: Chemistry(2019),385(112049).
**Response:**

Thanks for the reviewer’s good indication and suggestion. Based on your comments, we read these papers carefully and found their works were helpful for ours, so we have cited them as reference as [8], [9], [13], [14], [17], [20], [22]. The revised content is located on the page 3 line 6, 14, 20, 22 and page 4 line 1 and the revised part has been marked in red, which expressed as: Some photocatalysts have the function of degrading pollutants while Excellent antibacterial activity [8-9]. Due to its advantages of low toxicity, low preparation cost and high stability, it has been applied to the removal of organic pollutants in water, which has aroused extensive research interest [13-14]. Therefore, various methods have been evolved to enhance the photocatalytic activity of pure g-C_3_N_4_, including metal deposition [17-18], nonmetal doping [19], coupling with other materials [20], and using nano-sized structures [21]. By coupling g-C_3_N_4_ with other semiconductors to form a heterojunction structure, the shortcomings of high recombination rate of photogenerated electron-hole pairs of a single photocatalyst could be solved [22]. Unfortunately, one of the papers you recommended has not been found after our multiple searches (International Journal of Hydrogen Energy, doi:10.1016/j.ijhydene.2019.06.06.). Please re-confirm whether the corresponding journal or doi is correct.

**Comment 2:**

-There are various typo errors throughout the manuscript, for instance in line no. 4 “,” should be removed after MoO_3_, line no. 20 “.” should be checked for pg no. 8 and similarly for whole manuscript.

**Response:**

Thanks for your indication and suggestion. We apologize for our negligence, we have checked many times to ensure that similar mistakes will not occur.

**Comment 3:**

-Author has reported the effect of weight percent of MoO_3_ present in the composite but no evidence has been provided to confirm the composite elaborated weight composition of final ternary composite, therefore TGA should be done to support the stated fact.

**Response:**

Thanks for the reviewer’s good evaluation and kind suggestion. Based on your opinion, the TGA experiment is supplemented in our revised MS, and the weight percentage of each component in the composite material is calculated and analyzed. We rewritten the relevant content as: Thermo-gravimetric analysis (TGA) was carried out on a STA 449F3 thermal analyzer with a heating rate of 10 ^o^C/min from room temperature to 1000 ^o^C in an air flow. Fig. S2 displays TGA curves for the g-C_3_N_4_ and Fe_3_O_4_/g-C_3_N_4_/MoO_3_ (30%) samples. As can be seen, the pristine g-C_3_N_4_ shows a weight loss of about 96% after heating up to 750 ^o^C. Hence, it was concluded that the g-C_3_N_4_ decomposes almost completely heating up to 750 ^o^C. It is evident that the thermal behavior of Fe_3_O_4_/g-C_3_N_4_ and Fe_3_O_4_/g-C_3_N_4_/MoO_3_ (30%) samples are similar to that of g-C_3_N_4_. As can be seen, by loading Fe_3_O_4_ and MoO_3_ on the g-C_3_N_4_ sheets, thermal degradation of the nano-composites starts from lower temperature relative to the pristine g-C_3_N_4_. Hence, similar to many g-C_3_N_4_-based nanocomposites, thermal stability of the pristine g-C_3_N_4_ decreases with depositing different particles [45-46]. The g-C_3_N_4_ contents of Fe_3_O_4_/g-C_3_N_4_ and Fe_3_O_4_/g-C_3_N_4_/MoO_3_ (30%) nanocomposites were calculated from the weights remaining after heating the samples to over 650 ^o^C. The g-C_3_N_4_ contents of the Fe_3_O_4_/g-C_3_N_4_/MoO_3_ (30%) nanocomposite was about 8.2%, respectively. As can be seen, besides the weight loss of g-C_3_N_4_, another weight loss between 750 and 1000 ^o^C in the Fe_3_O_4_/g-C_3_N_4_/MoO_3_ (30%) composites, could be ascribed to the vaporization of MoO_3_. The MoO_3_ contents of the Fe_3_O_4_/g-C_3_N_4_/MoO_3_ (30%) is about 16.4 %. In addition, after calculation, The MoO_3_ contents of the Fe_3_O_4_/g-C_3_N_4_/MoO_3_ (30%) is about 75.4 %. The results were listed in Tab. 1, which were marked in red from page 7 line 17, page 12 line 21,The results are shown in Fig. S2 and Tab. 1.

Fig. S2 TGA curves of pure g-C_3_N_4_ and Fe_3_O_4_/g-C_3_N_4_/MoO_3_ (30%) photocatalysts.

| Compound | Weight percentage |
| --- | --- |
| g-C_3_N_4_ | 8.2 |
| MoO_3_ | 16.4 |
| Fe_3_O_4_ | 75.4 |

Tab. 1. Weight percentages of different compounds in the Fe_3_O_4_/g-C_3_N_4_/MoO_3_ (30%)nanocomposite.

**Comment 4:**

-Author should mention the complete details of photocatalytic instrument taken in use for the degradation studies like its capacity, the distance of light source from the surface of pollutant and light source intensity etc.

**Response:**

Thanks for the reviewer’s good evaluation and kind suggestion. According to your opinion, the complete details of the photocatalytic instrument for degradation research, such as its capacity, the distance between the light source and the surface of the pollutant, and the intensity of the light source are supplemented in our photocatalytic experimental design section. The result was displayed in line 1 page 8 in the MS, which expressed as: The capacity of the synthesized catalysts to photodegrade TC was performed by a photochemistry reaction instrument (YM-GHX-V, Shanghai Yuming Instrument Co. Ltd, China) with a 1000 W Xe lamp applied as visible light source, as shown in Fig. S1. In the reaction system, the reaction solution is packed in a quartz tube with a capacity of 50 ml, and the quartz tube is fixed at a distance of 2 cm from the light source. An optical power meter (OPT-1A, China) was used to measure the intensity of the experimental lamp to be 37.5 mW/cm^2^ (λ >400 nm). A water circulation system was utilized to keep the reaction system at 15 ^o^C. In each experiment, 10 mg of the photocatalyst was added into 50 mL of TC solution (40 mg/L).

Fig. S1 Schematic diagram of photocatalytic reaction device.

**Comment 5:**

-XRD patterns graph should be expressed more clearly, the peaks of g-C_3_N_4_ in 10% and g-C_3_N_4_/Fe_3_O_4_ composite is not clearly visible in present graph style.

**Response:**

Thanks for your indication and suggestion. According to your suggestion, in order to clearly express the XRD spectrum, we have redrawn the XRD spectrum, in which the peaks of g-C_3_N_4_ in 10% and g-C_3_N_4_/Fe_3_O_4_ composite is obvious.

Fig. 1. XRD patterns for the MoO_3_, g-C_3_N_4_, Fe_3_O_4_, Fe_3_O_4_/g-C_3_N_4_ and Fe_3_O_4_/g-C_3_N_4_/MoO_3_ nanocomposites.Inset image shows the deconvulation peaks for MoO_3_ and g-C_3_N_4_.

**Comment 6:**

-Authors must explain that how the compound weight percentage has been calculated from EDX data? EDX analysis can only give estimation for elemental composition and not compound in particular. The EDX analysis needs reconsideration.

**Response:**

Thank you for your kind suggestion. Based on your suggestions, we performed the TGA experiment, and re-evaluated the mass ratio of each compound based on TGA analysis. The results were list in Table 1. The EDX analysis were applied to display the elemental distributions. The related content was listed in line 6 page 10.

| Compound | Weight percentage |
| --- | --- |
| g-C_3_N_4_ | 8.2 |
| MoO_3_ | 16.4 |
| Fe_3_O_4_ | 75.4 |

Tab. 1. Weight percentages of different compounds in the Fe_3_O_4_/g-C_3_N_4_/MoO_3_ (30%)nanocomposite.

**Comment 7:**

**-**Fig 2(b,C) should also be represented with corresponding elements.

**Response:**

Thanks for your indication and suggestion. Based on your suggestions, we have re-marked Fig 2. We are very sorry for such mistakes.

Fig. 2. (a) SEM images of Fe_3_O_4_/g-C_3_N_4_/MoO_3_ (30%); (b-f) EDX mapping for the Fe_3_O_4_/g-C_3_N_4_/MoO_3_ (30%) nanocomposite.

**Comment 8:**

-As mentioned “Pure g-C_3_N_4_ (Fig. 3(b)) consisted of a transparent sheet structure” is not appearing as explained in TEM image. In image 3b the sheet like structure is not visible therefore need explanation or replacement.

**Response:**

Thanks for the reviewer’s good evaluation and kind suggestion. Based on your suggestions, we have replaced the “transparent sheet structure” in the analysis of the TEM image with “lamellar-like and smooth morphology”. This modified part is located on line 10 of page 17, and has been marked in red.

**Comment 9:**

The language should be improved throughout the manuscript.

**Response:**

Thank you for your kind suggestion. Based on your opinion, we have further improved the fluency of the sentences in the manuscript to make it easier to read and understand.

Thanks very much again for your comments and suggestions to our manuscript. We have tried our best to improve the manuscript and made some changes in the manuscript. We sincerely hope the revised manuscript can meet the requirements of yours.

Yours sincerely,

Yaohui Wu
